# Significant compositional and functional variation reveals the patterns of gut microbiota evolution among the widespread Asian honeybee populations

**DOI:** 10.3389/fmicb.2022.934459

**Published:** 2022-09-02

**Authors:** Qinzhi Su, Min Tang, Jiahui Hu, Junbo Tang, Xue Zhang, Xingan Li, Qingsheng Niu, Xuguo Zhou, Shiqi Luo, Xin Zhou

**Affiliations:** ^1^College of Food Science and Nutritional Engineering, China Agricultural University, Beijing, China; ^2^Department of Entomology, College of Plant Protection, China Agricultural University, Beijing, China; ^3^Key Laboratory for Bee Genetics and Breeding, Jilin Provincial Institute of Apicultural Sciences, Jilin, China; ^4^Department of Entomology, University of Kentucky, Lexington, KY, United States

**Keywords:** gut microbiota, Asian honeybee, population variation, pollen, nectar

## Abstract

The gut microbiome is a crucial element that facilitates a host’s adaptation to a changing environment. Compared to the western honeybee *Apis mellifera*, the Asian honeybee, *Apis cerana* populations across its natural range remain mostly semi-feral and are less affected by bee management, which provides a good system to investigate how gut microbiota evolve under environmental heterogeneity on large geographic scales. We compared and analyzed the gut microbiomes of 99 Asian honeybees, from genetically diverged populations covering 13 provinces across China. Bacterial composition varied significantly across populations at phylotype, sequence-discrete population (SDP), and strain levels, but with extensive overlaps, indicating that the diversity of microbial community among *A. cerana* populations is driven by nestedness. Pollen diets were significantly correlated with both the composition and function of the gut microbiome. Core bacteria, *Gilliamella* and *Lactobacillus* Firm-5, showed antagonistic turnovers and contributed to the enrichment in carbohydrate transport and metabolism. By feeding and inoculation bioassays, we confirmed that the variations in pollen polysaccharide composition contributed to the trade-off of these core bacteria. Progressive change, i.e., nestedness, is the foundation of gut microbiome evolution among the Asian honeybee. Such a transition during the co-diversification of gut microbiomes is affected by environmental factors, diets in general, and pollen polysaccharides in particular.

## Introduction

The gut microbiome often serves as a critical component in the host’s adaptation to a changing environment (Suzuki and Ley, 2020). Gut microbiota can benefit host animals in nutrition provision, pathogen resistance, and modulations of development and behavior (Cryan and Dinan, 2012; Engel and Moran, 2013; Kamada et al., 2013; Sommer and Bäckhed, 2013). On the other hand, gut microbiota may be shaped by the host’s adjustments to changing environments, such as range expansion accompanied by diet shifts (Baldo et al., 2015; Michel et al., 2018). In particular, for widespread species found in a large geographic range, environmental heterogeneity is expected to influence their gut microbiota (Yatsunenko et al., 2012; Henderson et al., 2015). This is because the geographic location of animal populations is linked with varied host genetics, local vegetation, and environmental microbe sources.

Studies based on *Apis mellifera* have established the framework for honeybee gut microbiota, revealing their essential role in the biology of the honeybee, such as facilitating pollen digestion (Engel et al., 2012; Zheng et al., 2019), host development (Zheng et al., 2017), and pathogen resistance (Kwong et al., 2017a; Wu et al., 2020). The species of honeybees each maintain a relatively simple but stable gut microbiota, comprising 5–9 core bacteria (>95% of total abundance) from phyla Proteobacteria, Firmicutes, and Actinobacteria (Kwong and Moran, 2016; Kwong et al., 2017b). Ancestry reconstruction of these core microbes suggested that they have probably become part of the symbiont system in the common ancestry of all extent corbiculate bees (Kwong et al., 2017b). Interestingly, although the honeybees share much of the core microbes at the phylotype level, each host species possesses a species-specific microbial community (Kwong et al., 2017b), with most core microbes showing distinct strain diversities among hosts, e.g., between *Apis mellifera* and *Apis cerana* (Ellegaard et al., 2020). However, little is known about how these gut symbionts have evolved within their hosts.

Among the different honeybee species, both the western (*A. mellifera*) and eastern honeybees (*A. cerana*) are widely distributed across tropical and temperate climates, each with endemic populations adapted to local habitats (Wallberg et al., 2014; Ji et al., 2020). Compared to *A. mellifera, A. cerana* populations across its natural range (much of eastern, southern, and southeastern Asia) (Radloff et al., 2010) remain mostly semi-feral and are less affected by bee management, which provides a good system to investigate how gut microbiota evolve under environmental heterogeneity on large geographic scales. However, investigations based on 16S rRNA did not provide sufficient resolution to differentiate tropical *A. cerana* populations from those of the temperate zones (Kwong et al., 2017b). There is still a knowledge gap on the biogeographical variation of gut microbes among *A. cerana* populations.

Our recent work on the evolution of mainland *A. cerana* revealed that multiple peripheral subspecies had radiated from a common central ancestral population and adapted independently to diverse habitats (Ji et al., 2020). During the most recent radiation period (∼100 ka), selective pressures imposed by diverse habitats, especially those of the changing floras, led to the convergent adaptation of the honeybee, where genes associated with sucrose sensitivity and foraging labor division had been repeatedly selected (Ji et al., 2020). We hypothesized that the gut microbiota of *A. cerana* had also evolved along with host range expansion, subspecies differentiation, and habitat adaptation. In the present study, we aimed to understand the landscape of gut microbial diversity and function across geographic populations of mainland *A. cerana* with metagenome sequencing. We also examined the effects of host genetics and diet variation on the honeybee gut symbionts. In addition, we explored the adaptive mechanisms of the microbes in response to selective pressures.

## Materials and methods

### Sample collection

A total of 99 worker bees of *A. cerana* were obtained from inside the hives at 15 sites in 13 provinces of China (Hainan, Yunnan, Taiwan, Fujian, Jiangxi, Hunan, Tibet, Sichuan, Shaanxi, Gansu, Qinghai, Hebei, and Jilin), between April 2017 and January 2019. For each population, ≥5 gut samples were sequenced from at least two hives to represent the diversity of each population (Supplementary Table 1). We collected nurse bees based on their morphology (Seeley, 1982). The nurse bees are generally characterized by relatively lightened color and apparently intact hairs and wings. Bees that were newly emerged (with shiny hair and slow-moving capability) or aged (with visible wing wear and hair loss) were excluded from sampling. Our sampling covered the main natural distribution range of *A. cerana* in China, from 19.2°-43.5°N, 95.7°-128.7°E, representing drastically different altitudes (12-3,325 m, Supplementary Table 1). The guts (including the midgut and hindgut) were dissected from the abdomen and stored in 100% ethanol or directly frozen at −80°C. To preserve live gut bacteria for strain isolation, a subset of guts was suspended in 100 μl of 25% glycerol (v/v, dissolved in PBS buffer), homogenized, and then frozen at −80°C.

### Isolation, cultivation, and identification of gut microbe strains

The gut homogenates were plated on different cultivation media, respectively, for various honeybee gut bacteria following Engel et al. (2013), including heart infusion agar (HIA) with 5% (v/v) de-fibrinated sheep blood, Columbia agar with 5% (v/v) de-fibrinated sheep blood, De Man, Rogosa and Sharpe (MRS) agar, and trypticase–phytone–yeast (TPY) agar supplemented with 1% mupirocin. The plates were incubated at 35°C in a 5% CO_2_ or anaerobic atmosphere.

When bacterial colonies became visible on the plates, they were identified by sequences of their 16S rRNA gene. The isolates were picked and dissolved with H_2_O, then boiled at 100°C for 1 min, which was used directly as a DNA template in PCR. PCR amplicons were generated using the universal 16S primers 27F (5′-AGAGTTTGATCCTGGCTCAG-3′) and 1492R (5′-GGTTACCTTGTTACGACTT-3′) with 25 cycles of amplification (94°C for 30 s, 60°C for 40 s, and 72°C for 60 s) after an initial incubation for 1 min at 95°C. Amplicons were sequenced using Sanger sequencing and identified using blastn against annotated sequences in GenBank.

### DNA extraction for genome and metagenome sequencing

The gut DNA was extracted following Kwong et al. (2017b). Briefly, the crushed gut was suspended in a capped tube with 728 μl of CTAB buffer, 20 μl of proteinase K, 500 μl of 0.1-mm Zirconia beads (BioSpec), 2 μl of 2-Mercaptoethanol, and 2 μl of RNase A cocktail. The mixtures were bead-beaten for 2 min for 3 times. After digested overnight at 50°C, the mixtures were added with 750 μl of phenol/chloroform/isoamyl alcohol (25:24:1, pH 8.0) and centrifuged to obtain the aqueous layer. After being precipitated at −20°C, spun at 4°C, and washed with −20°C ethanol, the DNA pellets were dried at 50°C, and then re-suspended in 50 μl of nuclease-free H_2_O. Final DNA samples were stored at −20°C.

Genomic DNA of honeybee gut bacterial isolates was also extracted using the phenol-chloroform protocol. The bacterial cells were re-suspended in 500 μl of lysis buffer [50 mM Tris-HCl (pH 8.0), 200 mM NaCl, 20 mM EDTA, nuclease-free H_2_O, 2% SDS, proteinase K (20 mg/ml)], then added with 500 μl of CTAB extraction buffer [50 mM Tris-HCl (pH 8.0), 20 mM EDTA, 1.4 M NaCl, 2% CTAB, 1% PVP 40000, nuclease-free H_2_O; pre-heated at 56°C]. The mixtures were incubated for 30 min at 65°C before the addition of 500 μl of phenol/chloroform/isoamyl alcohol (25:24:1, pH 8.0). Then, the mixture was centrifuged at 14,000 *g* at room temperature (RT) for 5 min. The aqueous layer was transferred to a new tube, added with 5 μl of RNase (100 mg/ml), incubated at RT for 20 min, and added with 600 μl of chloroform: isoamyl alcohol (24:1). After spinning at 14,000 *g* at RT for 5 min, the aqueous layer was transferred to a new tube and added with 5 μl of ammonium acetate (final concentration 0.75 M), 1 μl glycogen solution (20 mg/ml), and 1 ml of cold 100% ethanol. DNA was precipitated at −20°C for 30 min. Precipitations were spun at 14,000 *g* at 4°C for 15 min, and the supernatant was decanted. DNA pellets were washed with 80 and 70% ethanol pre-cooled at −20°C, respectively, and spun for an additional 10 min at 4°C. The supernatant was discarded and the DNA pellet was air dried. The pellet was re-suspended in 50 μl nuclease-free H_2_O and kept at 4°C overnight before being stored at −20°C.

### Genome and metagenome sequencing

A total of 99 honeybee gut samples were used for metagenome sequencing (Supplementary Table 1). In total, 83 representative core bacterial strains obtained from *A. cerana* were selected and sequenced to construct a reference genome library for phylotype, SDP, and single nucleotide variation (SNV) analyses (Supplementary Table 2). DNA samples were paired-end sequenced at the Beijing Genomics Institute Shenzhen Branch (BGI-Shenzhen) using the BGISEQ-500 platform (200–400 bp insert size; 100 bp read length; paired-ended [PE]) and at Novogen company using the Illumina Hiseq X Ten platform (350 bp insert size; 150 bp read length; PE). One *Gilliamella* strain (B3022) was sequenced on the PacBio RS II platform at NextOmics company.

### Bacterial genome assembly and annotation

Low-quality reads from the Illumina Hiseq X Ten platform were filtered out using fastp (Chen et al., 2018) (version 0.13.1, -q 20 -u 10) before subsequent analyses. For isolated bacterial strains, clean data were assembled using SOAPdenovo (Luo et al., 2012) (version 2.04, -K 51 -m 91 –R for PE 150 reads; -K 31 -m 63 –R for PE 100 reads), SOAPdenovo-Trans (Xie et al., 2014) (version 1.02, -K 81 -d 5 -t 1 -e 5 for PE 150 reads; -K 61 -d 5 -t 1 -e 5 for PE 100 reads), and SPAdes (Bankevich et al., 2012) (version 3.13.0, -k 33,55,77,85) based on contigs assembled by SOAPdenovo (only for PE 150 reads) or SOAPdenovo-Trans. The assembly with the longest N50 was retained for each strain as the draft genome. Then clean reads were mapped to the assembled scaftigs using minimap 2-2.9 (Li, 2018) and the bam files were generated by samtools (Li H. et al., 2009) (version 1.8). Genome assemblies were processed by BamDeal^1^ (version 0.19) to calculate and visualize the sequencing coverage and GC content of the assembled scaftigs. Scaftigs with aberrant depths and GC contents were then removed from the draft genome. Next, the remaining scaftigs were filtered taxonomically. Scaftigs assigned to eukaryote by Kraken2 (Wood et al., 2019) using the standard reference database were removed, and the ones aligned to a wrong phylum by blastn (megablast with *e* < 0.001) were further removed. The remaining genome assemblies were used as bacterial genome references. The *Giliamella* strain (B3022) sequenced on the PacBio RS II platform was assembled using a hierarchical genome assembly method (HGAP2.3.0) (Chin et al., 2013).

The protein coding regions of bacterial genomes were predicted using Prokka version 1.13 (Seemann, 2014). The KEGG orthologous groups (KOs) annotation was carried out using KofamKOALA (Aramaki et al., 2020) based on profile HMM and adaptive score threshold with default parameters. Programs KEGG Pathway and Brite Hierarchy were used to screen the annotation results. Finally, dbCAN2 version 2.0.11 (Zhang et al., 2018) was applied to annotate CAZymes and CAZyme gene clusters (CGCs) using embedded tools HMMER, DIAMOND, and Hotpep with default parameters.

### Genetic variation of *A. cerana* hosts

Metagenomes were filtered by fastp (-q 20 -u 10) (Chen et al., 2018). Clean reads were then mapped to the *A. cerana* reference genome (ACSNU-2.0, GCF_001442555.1) (Park et al., 2015) using the BWA-MEM algorithm (v 0.7.17-r1188) (Li and Durbin, 2010), with default settings and an additional “-M” parameter to reach compatibility with Picard. Read duplicates were marked using Picard MarkDuplicates 2.18.9^2^. GATK HaplotypeCaller in the GVCF mode (McKenna et al., 2010) (v4.0.4) was used to call variants for each sample. All of the per-sample GVCFs were joined using GenotypeGVCFs. Then, the final variant file retained SNPs that met all of the following criteria: (1) average depth >5× and <40×; (2) quality score (QUAL) > 20; (3) average genotype quality (GQ) > 20; (4) minor allele frequency (MAF) > 0.05; (5) proportion of missing genotypes < 50%; and (6) bi-allelic SNP sites.

The identity by state (IBS) distance matrices were performed and constructed with the filtered SNPs using functions “snpgdsIBS” in the R package SNPRelate (Zheng et al., 2012). A neighbor-joining tree was reconstructed based on the IBS distance matrix using the function “nj” in the R package Ape (Paradis et al., 2004). Node support values were obtained after 1,000 bootstrap replicates.

### Reference-based metagenome composition analyses

Shotgun reads generated from the whole honeybee gut were first mapped against the *A. cerana* genome (ACSNU-2.0, GCF_001442555.1) using BWA aln (version 0.7.16a-r1181, -n 1) (Li and Durbin, 2010) to identify host reads, which were subsequently excluded. For taxonomic assignments of bacterial sequences, we used Kraken2 (Wood et al., 2019) and Bracken version 2.0 (Lu et al., 2017) to profile bacterial phylotype composition and used MIDAS (Nayfach et al., 2016) to profile strain composition for metagenomic samples. The reference database contained 390 bacterial genomes, including 307 published genomes and 83 newly-sequenced *A. cerana*-derived strains from this study (Supplementary Table 2). The majority of the reference strains belonged to six core phylotypes (*Gilliamella, Snodgrassella, Bifidobacterium, Lactobacillus* Firm-4, *Lactobacillus* Firm-5, and *Apibacter*) of honeybee gut bacteria. The analyses of public gut metagenome data of *A. cerana* from Japan (Ellegaard et al., 2020) and *A. mellifera* (Ellegaard and Engel, 2019; Ellegaard et al., 2020) followed the same pipeline.

### Identification and profiling of sequence-discrete population

We defined SDPs for each core gut bacterium (*Gilliamella*, *Snodgrassella*, *Bifidobacterium*, *Lactobacillus* Firm-4, *Lactobacillus* Firm-5, and *Apibacter*) using a 95% gANI threshold (Richter and Rosselló-Móra, 2009). Pairwise average nucleotide identities were calculated using the pyani Python3 module^3^. To generate the whole-genome tree for each core bacterium, we used Roary version 3.12.0 (Page et al., 2015) with the parameter -blastp 75 to obtain core single-copy genes shared among all strains. The alignments of nucleotide sequences were concatenated, from which a maximum-likelihood tree was inferred using FastTree version 2.1.10 (Price et al., 2010) with a generalized time-reversible (GTR) model and then visualized using iTOL (Letunic and Bork, 2019).

We used the ‘run_midas.py species’ script of MIDAS (Nayfach et al., 2016) with default parameters to estimate SDP relative abundances in each sample. The script ‘merge_midas.py species’ with the option ‘–sample_depth 10.0’ was used to merge SDP abundance files across samples. The SDPs with a relative abundance of less than 1% were filtered out.

### Detection of single nucleotide variation and copy number variations across populations

CheckM version 1.0.86 (Parks et al., 2015) was used to estimate the completeness and contamination of genomes. The genome with the highest completeness and lowest contamination was selected as the reference sequence for each SDP. The metagenomic reads were mapped against reference genomes and the SNVs were quantified along the entire genome using MIDAS (Nayfach et al., 2016) and the script ‘run_midas.py snps’ with default parameters. For each SDP, the script ‘merge_midas.py snps’ pooled data across multiple samples with options ‘–snp_type bi –site_depth 5 –site_prev 0.05 –sample_depth 5.0 –fract_cov 0.4 –allele_freq 0.01’ to obtain the minor allele (second most common) frequency file. Thus, bi-allelic SNVs prevalent in more than 5% of profiled samples were predicted and rare SNVs with abnormally high read depth were excluded. The matrix files of SNVs remaining polymorphic were obtained after filtering steps.

We used the ‘run_midas.py genes’ script in MIDAS (Nayfach et al., 2016) to map metagenomic reads to pangenomes of each SDP and quantified gene copy numbers with default parameters. Then, we merged results from pangenome profiling across samples with the option ‘–sample_depth 5.0’ from the ‘merge_midas.py genes’ module. The gene coverage was normalized by the coverage of a set of 15 universal marker genes to obtain the estimated copy number for genes of each SDP. The coverage of each KO term was obtained by summing up all genes annotated as the same KO for each SDP. *p*-values were calculated using the Kruskal–Wallis one-way analysis across populations with the ‘compare_means’ function in the R package ‘ggpubr.’ KO copy number variation and SNV of each SDP were detected as highly variable when an adjusted *p* < 0.05.

### *De novo* assembly of metagenomes

The metagenome was also *de novo* assembled using MEGAHIT (Li et al., 2016) (version 1.1.2, -m 0.6 –k-list 31,51,71 –no-mercy) for each gut sample. Assemblies longer than 500 bp were blasted against the NCBI nr database using DIAMOND (Buchfink et al., 2015) (version 0.9.22.123, blastx -f 102 -k 1 -e 1e-3) and were assigned to fungi, bacteria, archaea, virus, or plants (Viridiplantae). Only assemblies assigned as bacteria were retained for further analyses.

A customized bacterial genome database was constructed to enable taxonomic assignments for the bacterial assemblies. The database included all bacterial genomes available on NCBI^4^ up to Jan 2019 (167,172 genomes), 83 genome assemblies of newly sequenced *A. cerana* gut bacteria (Supplementary Table 2), and 14 *Apibacter* genomes from *A. cerana* (Zhang et al., 2022). Taxonomical assignments were conducted using blastn, and an e-value of 1e-5 was observed. The assemblies were assigned to the genus of the best hit, while those without any hits were defined as unassigned bacteria.

For each metagenome sample, all clean reads were mapped against bacterial assemblies using SOAPaligner (Li R. et al., 2009) (version 2.21, -M 4 -l 30 -r 1 -v 6 -m 200). The results were summarized using the soap.coverage script (version 2.7.7^5^). Only assemblies with ≥90% coverage were considered true bacteria. Shannon index and Bray–Curtis dissimilarity were calculated using the vegan R package (Philip, 2003). The analyses of public gut metagenome data of *A. cerana* from Japan (Ellegaard et al., 2020) and *A. mellifera* (Ellegaard and Engel, 2019) followed the same pipeline.

### Gene prediction and functional annotation for metagenomes

Gene prediction was conducted using MetaGeneMark (Zhu et al., 2010) (GeneMark.hmm version 3.38) with the *de novo* metagenome assemblies, and those longer than 100 bp were clustered using CD-HIT (Li and Godzik, 2006) (version 4.7, -c 0.95 -G 0 -g 1 -aS 0.9 -M 0) to obtain a non-redundant gene catalog for *A. cerana* metagenomes. For each individual metagenome sample, clean data were aligned onto the non-redundant gene catalog using SOAPaligner (Li R. et al., 2009) (version 2.21, -M 4 -l 30 -r 1 -v 6 -m 200). The gene abundance was calculated using soap.coverage script (version 2.7.9, see Text Footnote 5). For each sample, only assemblies of ≥90% coverage were retained for further annotation. The analyses of public gut metagenome data of *A. cerana* from Japan (Ellegaard et al., 2020) and *A. mellifera* (Ellegaard and Engel, 2019) followed the same pipeline.

Functional annotation of the gene catalog was performed by GhostKOALA (Kanehisa et al., 2016) using the genus_prokaryotes KEGG GENES database and KofamKOALA (Aramaki et al., 2020) with an e-value threshold of 0.001. Genes were first assigned with KO ID predicted by KofamKOALA, and the remaining unassigned genes were then annotated using GhostKOALA. KOs were mapped onto KEGG pathways using the KEGG Mapper online^6^.

The abundances of KOs and pathways were calculated as the sum of the abundances of all genes annotated to them using custom scripts. Population dissimilarities (Bray–Curtis distance) of KO function among the 15 bee populations were tested by the ANOSIM test included in the vegan package (Philip, 2003) with 999 permutations. Linear discriminant analysis (LDA) was performed using LEfSe (Segata et al., 2011) with default parameters to identify KO biomarkers in different populations. Function enrichment of featured KOs was estimated by one-sided Fisher’s Exact Test using the stats R package at both module and pathway levels.

For each featured KO, the abundances for all bacterial species encoding the KO-related genes were listed for all of the 99 samples. In each population, the median abundance was used as the abundance of bacterial species encoding the respective KO. Then, the contributions by different bacterial species to the corresponding KO were estimated. If the KO term was identified in > 50% of individual bee guts of the same population, the KO was considered to be present in the population. To compare the gene numbers among different populations, we standardized metagenome data by randomly extracting 400 Mb bacterium-derived data from each gut sample, which were mapped to the gene assemblies. The assemblies were retained only if the coverage was ≥90%.

The glycoside hydrolase (GH) and polysaccharide lyase (PL) genes were functionally assigned to the dbCAN2 database (Zhang et al., 2018). In each population, the median abundance was used as the abundance of bacterial species encoding respective GH/PL gene clusters. Then, the contributions by different bacterial species to the corresponding GH/PL gene clusters were estimated.

### Diet profiling of gut and honey metagenomes

A customized chloroplast genome database was first constructed for flowering plants (4,161 from NCBI and 271 newly sequenced ones generated by our group) for KrakenUniq version 0.5.5 (Breitwieser et al., 2018). For gut metagenome data, we filtered out reads mapped to the *A. cerana* genome or to the *de novo* bacterial assemblies and used the remaining reads for pollen diet profiling based on chloroplast DNA found in the gut annotated at the family level. The remaining reads were first aligned to the customized chloroplast genomes with KrakenUniq (Breitwieser et al., 2018) with default parameters. Those mapped reads were aligned to nt database with blastn with an e-value setting as 1e-5, and the best alignment was retained. Then, the reads from the alignments with similarity >95% and query coverage >90% to reference sequences from plants were kept and used to estimate the pollen abundance at the family level. The families with a relative abundance of less than 1% were filtered out.

The geographical variation in pollen composition was also conducted with the assembled metagenome data from honey samples collected from five representative regions of this study (SC_AB, SC_GB, SX_QL, QH_GD, JL_DH) (Liu et al., 2021). The assemblies with similarity >95% and query coverage >90% to reference plant sequences were retained. Clean reads were then aligned to these assemblies using Minimap2 (Li, 2018), and the mapped reads were used to estimate the pollen abundance at the family level with SamBamba (Tarasov et al., 2015). The families with a relative abundance of less than 1% were filtered out.

The gut bacterial phylotype and KO composition from *de novo* assembly and annotation were used in the correlation analysis with pollen composition at the family level.

### Heritability of bacterial diversity

The rank-based inverse normal transformation of the relative abundance with the reference-based method was used in the heritability analysis. The heritability was defined as the Percentage of Variance Explained (PVE) and estimated with Genome-wide Efficient Mixed Model Association (GEMMA, v0.94) (Zhou and Stephens, 2012). To control the effects of individual relatedness, population structure, and diet variation, we regressed the transformed gut bacteria abundance with the first three PCs from the PCA of the host genotypic data and the pollen Shannon index from the gut. Then, PVE estimation was performed with the residuals using GEMMA (with relatedness matrices and the HE regression algorithm). A phylotype or SDP was considered heritable if the PVE measurements did not show overlaps with zero.

### The association between host genetic variation and bacterial diversity

The rank-based inverse normal transformation of the relative abundance of core gut bacteria was used in the Genome-Wide Association Studies (GWAS) analysis. We used the Linear Mixed Model in rMVP v1.0.0 (Yin et al., 2021). In the GWAS analysis, the kinship between individuals, the first three PCs in host PCA, and the diet (Shannon index of pollen family composition) were used for correction. We used the ‘EMMA’ method to analyze variance components in rMVP. The statistical significance level was set to *p* < 5 × 10^–8^ for the GWAS association.

### The effects of diet on the abundance of *Gilliamella* and *Lactobacillus* Firm-5

A *Gilliamella* strain (B2889, belonging to the SDP Gillia_Acer_2) was cultivated with HIA, and a *Lactobacillus* Firm-5 strain (B4010) was cultivated with MRS with 0.02 g/ml D-fructose (aladdin F108331) and 0.001 g/ml L-cysteine (aladdin C108238). The microbiota-free *A. cerana* workers were obtained following Zhang et al. (2022). Pupae in the late stage were removed from brood frames and incubated in sterile plastic bins at 35°C. Both bacterial strains of OD_600_ = 1 were mixed at equal volumes and then mixed with 50% (v/v) sterilized sucrose syrup, which was fed to newly emerged microbiota-free honeybees. After 3 days, cellobiose (Shanghai Yuanye Bio-Technology Co., Ltd. S11030, final concentration 5 mg/ml) and solutions with different proportions of pectin (Sigma, P9135) and cellulose (Megazyme, P-CMC4M) (1:10, 10:1, final mixed concentration 5.5 mg/ml) mixed with sterilized 50% sucrose syrup were fed to honeybees, respectively. Honeybees fed with only 50% sucrose syrup were used as control. After feeding for 4 days, DNA was extracted from bee guts and used for the qPCR assay.

### qPCR assay

We conducted real-time qPCR experiments to determine bacterial loads for both *Gilliamella* and *Lactobacillus* Firm-5 after the feeding experiments. 16S-F-Gillia (5′-TGAGTGCTTGCACTTGATGACG-3′) and 16S-R-Gilla (5′-ATATGGGTTCATCAAATGGCGCA-3′) primers were used for *Gilliamella* 16S rRNA gene amplification. 16S-F-Firm5 (5′-GCAACCTGCCCTWTAGCTTG-3′) and 16S-R-Firm5 (5′-GCCCATCCTKTAGTGACAGC-3′) primers (Kešnerová et al., 2017) were used for *Lactobacillus* Firm-5 16S rRNA gene amplification. Actin-AC-F (5′-ATGCCAACACTGTCCTTTCT-3′) and Actin-AC-R (5′-GACCCACCAATCCATACGGA-3′) primers were used to amplify the *actin* gene of the host *A. cerana* (Park et al., 2020), which was used to normalize the bacterial amplicons (Kešnerová et al., 2017). The 16S target sequences were cloned into vector pEASY-T1 (Transgen), and the Actin target sequence was cloned into pCE2 TA/Blunt-Zero Vector (Vazyme), then confirmed by Sanger sequencing. The copy number of the plasmid was calculated, serially diluted, and used as the standard. qPCR was performed using the ChamQ Universal SYBR qPCR Master Mix (Vazyme) and QuantStudio 1 (Thermo Fisher) in a standard 96-well block (20-μl reactions; incubation at 95°C for 3 min, 40 cycles of denaturation at 95°C for 10 s, and annealing/extension at 60°C for 20 s). The data were analyzed using the QuantStudio Design & Analysis Software v1.5.1 (Thermo Fisher) and Excel (Microsoft). *p*-values were calculated using the Mann–Whitney test.

## Results

### Bacterial composition significantly varied across Asian honeybee populations at multiple levels

**FIGURE 1:**
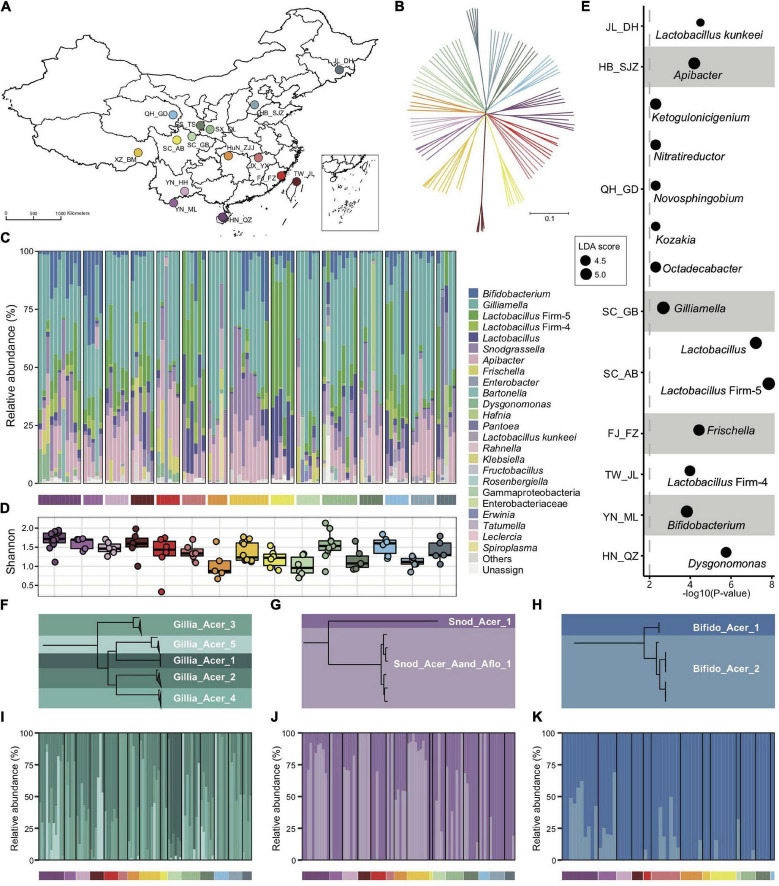
Bacterial composition of gut microbiota in geographic populations of *Apis cerana*. **(A)** Sampling sites of 15 *A. cerana* geographic populations. **(B)** Neighbor-joining tree reflecting the honeybee population structure, based on genome-wide SNPs. Bacterial relative abundance **(C)** and Shannon index **(D)** are based on gut metagenomes of different populations. Phylotypes with at least 5% abundance in any sample or 0.5% abundance in more than 6 samples were shown, otherwise included in “Others.” *Lactobacillus*: *Lactobacillus* that is not assigned to any known groups. **(E)** Featured gut microbe phylotypes in each geographic population were revealed by LEfSe analyses. The size of the bubbles represents LDA score. Phylogenetic relationships of SDPs within *Gilliamella* **(F)**, *Snodgrassella* **(G),** and *Bifidobacterium* **(H)**. Maximal-likelihood phylograms, reconstructed using core genes present in all strains of the corresponding phylotype. The SDP compositions of *Gilliamella* **(I)**, *Snodgrassella* **(J)**, and *Bifidobacterium* **(K)** in gut samples, with those of abundances <1% excluded. Horizontal bars under panels **(C,I–K)** indicate population origins of the guts, with colors corresponding to those in **(A,B)**.

A total of 99 nurse bees from 15 geographic populations covering 13 provinces across China were analyzed (Figure 1A and Supplementary Table 1). SNPs derived from honeybee reads were used to construct a neighbor-joining tree (Figure 1B), which confirmed the geographic origin of the sampled populations. This result was consistent with the reported genetic structure and geographic distribution of *A. cerana* populations (Ji et al., 2020), thereby excluding the possibility of colony translocation.

Bacterial reads were then *de novo* assembled and aligned against the GenBank nr database to recover the phylotype composition for individual nurse bees. In congruence with previous studies (Kwong et al., 2017b; Ellegaard et al., 2020), the core gut microbiota in *A. cerana* included six groups of bacteria, i.e., *Gilliamella* and *Snodgrassella* from Proteobacteria, *Bifidobacterium* from Actinobacteria, *Lactobacillus* Firm-4 and Firm-5 from Firmicutes, and *Apibacter* from Bacteroidetes (Figure 1C). This result was further confirmed by the reference-based method (Supplementary Figures 1, 2), which employed a customized database containing 307 public and 83 newly sequenced bee gut bacterial genomes (Supplementary Table 2). However, apparent variations in phylotype composition were observed among individual bees (Figure 1C), and the composition of core microbes appeared to be less stable than in *A. mellifera* (Regan et al., 2018; Ellegaard and Engel, 2019; Ge et al., 2021).

Both Shannon index (Figure 1D, Kruskal–Wallis, *P* = 0.0022) and phylotype diversity (ANOSIM, *r* = 0.29, *P* = 0.001) showed noticeable differences across populations of *A. cerana*. Nine of the 15 investigated populations could be defined by featured bacteria in the LEfSe analysis (Segata et al., 2011), which showed significantly higher relative abundances in the focal population than all remaining populations (Figure 1E).

The distinct gut variation across host populations was also reflected at finer taxonomic scales. Among all six core phylotypes in *A. cerana*, *Gilliamella* contained the most diverse host-specific sequence-discrete populations (SDPs) (Figures 1F–H and Supplementary Figures 3–6), which were defined as strains sharing a genome-wide average nucleotide identity (gANI) >95% within each phylotype. Our results revealed varied presence and abundance in SDPs of core phylotypes among gut samples (Figures 1I–K and Supplementary Figure 7), whereas *Gilliamella* showed significant SDP differences among geographical populations (Supplementary Figure 8, ANOSIM *r* = 0.14, *p* = 0.001). We also identified genome sites showing single nucleotide variation (SNV) for major SDPs in each sample, to detect gut variations at the strain level (Supplementary Figure 9). The results demonstrated significant variations in SNV composition across populations (Supplementary Figure 10). Thus, the gut bacterial composition of Asian honeybees varied significantly across geography at phylotype, SDP, and strain levels.

### Progressive changes in the honeybee microbial community were related to diet shift

**FIGURE 2:**
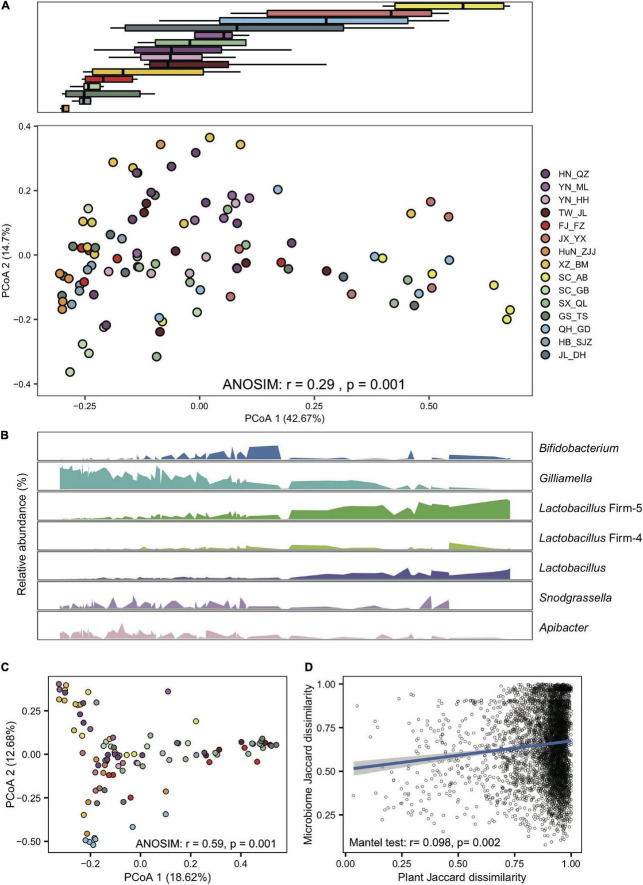
*Gilliamella* and *Lactobacillus* Firm-5 showed antagonistic trends in compositional turnover of honeybee gut microbiomes. **(A)** Overall variation of the gut microbial community at the phylotype level, revealed by Bray–Curtis dissimilarity PCoA (bottom panel). Boxplots (top panel) indicate the distribution of each population along the first principal coordinate axis (PCoA1). Boxplot center values represent the median and error bars represent the SD. Colors correspond to the population origin of the gut samples. **(B)** Relative abundances of core bacterial phylotypes along PCoA1. **(C)** The pollen composition at the family level varied in gut metagenomes from populations of *A. cerana*. **(D)** The Jaccard distances of the gut bacterial phylotype and the pollen composition at the family level were significantly correlated.

Gut compositions showed extensive overlaps among populations, forming continuous groups in PCoA analyses (Figure 2A), indicating progressive changes in microbial community structure among endemic honeybee populations. Interestingly, a continuous variable contributing to the separation along the first principal coordinate axis (PCoA) reflected antagonistic dynamics in abundances of *Gilliamella* and *Lactobacillus* Firm-5 (Figure 2B). Among all six core phylotypes, the relative abundance of *Gilliamella* (Spearman’s *rho* = −0.85, *p* = 2.14e-28) and *Lactobacillus* Firm-5 (Spearman’s *rho* = 0.79, *p* = 4.47e-22) showed the most significant correlation with the PCoA1 value.

In the 99 samples, the populations from XZ_BM, SC_AB, HN_QZ, JL_DH, TW_JL, and QH_GD represent each of the peripheral six subspecies, and the others are from the central ancestral population (Ji et al., 2020). We first tested whether the gut compositions showed differences at the subspecies level. We compared the Bray–Curtis distance between each of the central populations and between central and peripheral populations. The gut variations among subspecies were significantly more prominent than those within subspecies at the phylotype level (Wilcoxon test, *p* = 1.4e-7) but not at the SDP level (Wilcoxon test, *p* = 0.87). The results indicated that the gut microbiome changed along host differentiation, which might be related to the host genetic differentiation and diet variation.

Next, we estimated the heritability of the relative abundance of core bacteria at both phylotype and SDP levels. The heritability was overall low. Among the core phylotypes, *Gilliamella* abundance showed the highest heritability (Supplementary Figure 11), while that of *Snodgrassella* was not obvious. The abundances of about one-third SDPs were not heritable. The GWAS analysis did not detect any apparent site variation that had determined bacterial composition, as no genomic region of *A. cerana* was found significantly associated with the bacterial abundance (with a threshold as *p* < 2e-8) at either the phylotype or SDP level. These results indicated that gut microbial diversity at the population level was not likely driven by single-site nucleotide variations. The complex genetic heterogeneity and limited sample size might also mask the effect of host genetics.

To examine the effect of diet on the gut microbiome, we first extracted pollen reads from the metagenome data and identified flower composition for each gut sample (details in Section “Materials and methods”). Honeybee populations from different regions showed significant variation in pollen diet at the family level (ANOSIM, *r* = 0.59, *P* = 0.001, Figure 2C and Supplementary Table 3). Such a diet shift was further confirmed by pollen variation in honey samples extracted from five of the representative populations (SC_AB, SC_GB, SX_QL, QH_GD, and JL_DH), where pollen composition again showed significant differences at the family level (ANOSIM, *r* = 0.35, *p* = 0.007, Supplementary Figure 12 and Supplementary Table 4). Most importantly, the Jaccard distances of the gut bacterial phylotype and the pollen composition were significantly correlated (mantel test, *r* = 0.098, *p* = 0.002, Figure 2D). Among the core phylotypes, the abundances of *Gilliamella* showed a significant correlation with the Shannon index of pollen composition in the gut (Spearman’s *rho* = −0.23, *p* = 0.020). Therefore, the pollen diet showed a correlation with the composition of the honeybee gut microbiome.

### KEGG orthology function was correlated with diets and characterized by carbohydrate metabolism and transport

**FIGURE 3:**
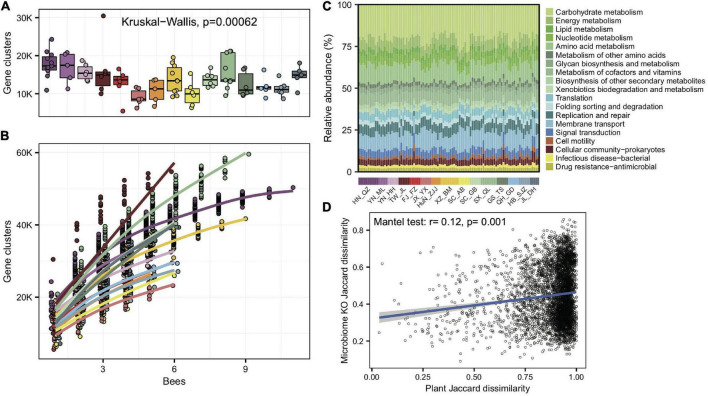
Significant variations in gene cluster and functional annotation among populations. **(A)** Gene cluster numbers per gut sample, based on 400 Mb bacteria-mapped reads. **(B)** Accumulation curves for gene clusters of each population of *A. cerana*, based on 400 Mb bacteria-mapped reads. **(C)** Relative abundance of KEGG annotations in each gut sample, based on all bacteria-mapped reads in metagenomes. **(D)** The Jaccard distances of the gut bacterial KO composition and the pollen composition at the family level were significantly correlated.

To understand whether gut microbes in *A. cerana* showed idiosyncratic regional traits on the function level, we *de novo* assembled the metagenomes and annotated genes for each of the 99 gut samples. As with bacterial compositions, the number of gene clusters per gut varied significantly among populations (Kruskal–Wallis test, *p* = 6.2e-4) (Figure 3A). The gene cluster number in different individuals was significantly correlated with the Shannon index of gut bacteria (Pearson’s *r* = 0.64, *p* = 8.28e-13), suggesting that bacterial diversity is the basis for gene varieties among individual bees. We also quantified the rate of novel gene accumulation for each population. The results demonstrated distinct differences in the genetic diversity among populations (Figure 3B).

We assigned predicted gene clusters from all metagenome data to the KEGG database to reveal the diversity of functions among populations. A total of 1,965 functional orthologs (KOs) shared among all populations were enriched in genetic information processing and signaling and cellular processes (Supplementary Figure 13). The KO category compositions (Figure 3C) also showed extensive overlap and were distinctively differentiated among populations (ANOSIM, *r* = 0.33, *p* = 0.001, Supplementary Figure 14). The LEfSe analyses showed that 11 of the 15 geographic populations had noticeably enriched KO categories (Supplementary Figure 15), which showed significantly higher relative abundances in the focal population than all remaining populations.

We also tested whether the KO compositions showed a difference at the subspecies level. The gut bacterial KO composition among subspecies was significantly more prominent than those within subspecies (Wilcoxon test, *p* = 1.6e-4). Furthermore, the Jaccard distances of the gut bacterial KO composition and pollen diversity at the family level showed a significant correlation (mantel test, *r* = 0.12, *p* = 0.001, Figure 3D). The results indicated that not only bacterial composition but also their functions changed along host differentiation and were associated with diets.

**FIGURE 4:**
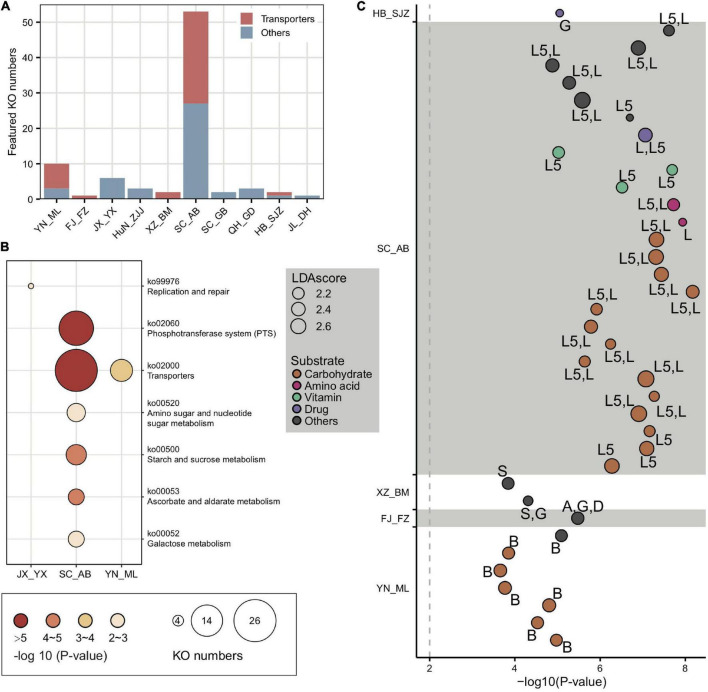
Locally featured KOs were enriched in carbohydrate transporters. **(A)** Featured KOs in geographic populations were enriched in transporters. **(B)** Featured KEGG pathways in gut microbiota from *A. cerana* populations. The size of the bubbles represents KO numbers. **(C)** Transporters in featured KOs were mainly specialized for carbohydrates. The size of the bubbles represents the LDA score. The codes marked next to each bubble indicate the main contributing bacteria species, where only those with >10% contribution were listed: A, *Apibacter*; B, *Bifidobacterium*; D, *Dysgonomonas*; G, *Gilliamella*; L, *Lactobacillus* that is not assigned to any known groups; L5, *Lactobacillus* Firm-5*;* S, *Snodgrassella*.

The top population-enriched KOs (*p* < 1e-5) mainly included functions in metabolism and membrane transport (Supplementary Figure 15). At the KO term level, we identified 83 KO terms showing inter-population differences (Supplementary Table 5), in which they were significantly more abundant in only one of the geographic populations. Interestingly, 37 of 83 of the enriched KO terms were transporter pathway genes (all belonging to ko02000) (Figure 4A), whereas the pathway was also enriched in some local populations (e.g., SC_AB and YN_ML, Figure 4B). Most featured transporters were related to carbohydrates (Figure 4C) and six of the enriched KO terms belonged to the glycoside hydrolase (GH) family (Supplementary Table 5), in concert with the fact that polysaccharides are one of the major nutritional components derived from pollen. Therefore, the population-enriched gut microbe KOs were mainly associated with carbohydrate metabolism and transport and were significantly correlated to pollen composition in a given local environment.

### Phosphotransferase system, ATP binding cassette transporters, and glycoside hydrolases contributed by *Gilliamella*, *Lactobacillus* Firm-5 and *Bifidobacterium* were hotspot genes involved in local adaptation

**FIGURE 5:**
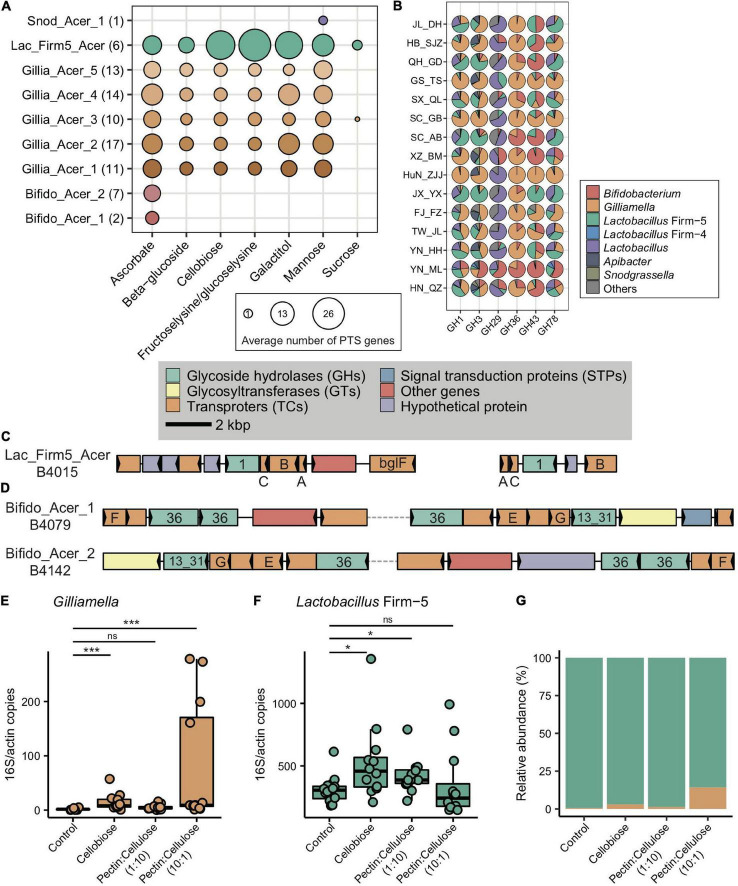
Main bacterial phylotypes coding for PTS and GHs. **(A)** Gene copy numbers in population-featured PTS pathways identified in all SDPs. Numbers in parentheses represent SDP strain numbers. Genes were annotated from the genomes of newly isolated microbial strains from *A. cerana* guts. **(B)** Featured GHs were coded by different bacterial phylotypes from metagenome of 15 geographic populations of *A. cerana*. **(C)** PTS transporters (*celA*/*celB*/*celC*/*bglF*), 6-phospho-beta-glucosidase (*bglA*) from the GH1 family were often found located together in genomes, which were represented here by *Lactobacillus* Firm-5 SDP. **(D)** ABC transporters (*msmE*/*msmF*/*msmG*), alpha-galactosidase from the GH36 family, and alpha-glucosidase from the GH13_31 family were often found located together in genomes, which were represented here by two *Bifidobacterium* SDPs. The change in the absolute abundance of *Gilliamella* **(E)**, *Lactobacillus* Firm-5 **(F)**, and the percentage of *Gilliamella* and *Lactobacillus* Firm-5 **(G)** after feeding cellobiose and mixtures of pectin and cellulose with different concentrations. PTS, phosphotransferase system; GH, glycoside hydrolase. ns, not significantly different, *p < 0.05, ***p < 0.001.

In congruence with the finding that carbohydrate metabolism and transport play important roles in adapting to local diets, key genes of such pathways, such as phosphotransferase system (PTS) transporters and ATP binding cassette (ABC), were often characterized in distinct honeybee populations. For instance, a total of 17 PTS and 16 ABC transporters were identified from the 37 enriched transporter pathway genes (Supplementary Table 5). All featured PTS genes were only found in the SC_AB population, while the featured ABC transporters were present in several populations (XZ_BM, SC_AB, and YN_ML). PTS serves as one of the major mechanisms in carbohydrate uptake, particularly for hexoses and disaccharides. In SC_AB, the 17 featured PTS genes included some that are specific for ascorbate, beta-glucoside, cellobiose, fructoselysine/glucoselysine, galactitol, mannose, and sucrose (Supplementary Table 5). The mapping of relevant gene clusters against the bacterial nr database suggested that these featured PTS genes were mainly contributed by *Gilliamella* and *Lactobacillus* Firm-5 (Supplementary Table 6). The dominant role of these two bacteria in coding PTS genes was further confirmed by analyses of 81 individually sequenced and annotated genomes, where *Gilliamella* and *Lactobacillus* Firm-5 were the major phylotypes encoding PTS genes (Supplementary Table 7). At the SDP level, *Lactobacillus* Firm-5 had a higher copy number of PTS transporters for cellobiose, fructoselysine/glucoselysine, and galactitol than *Gilliamella* (Figure 5A). Many of these PTS transporters were found in the featured genes in the SC_AB population, which was dominated by *Lactobacillus*. Thus, the enrichment of featured PTS genes could at least be partially explained by the elevated abundance of the contributing bacteria in local populations (Figure 1E).

The featured ABC transporters included transporters for amino acids, iron, and carbohydrates (Supplementary Table 5). Besides *Gilliamella* and *Lactobacillus* Firm-5, *Bifidobacterium* also contributed unique ABC transporters (Supplementary Table 6). For example, the *Bifidobacterium*-unique transporters for raffinose/stachyose/melibiose (msmE, msmF, and msmG) (genome annotation results in Supplementary Table 7) were featured in the YN_ML population, in which *Bifidobacterium* was also the featured phylotype (Figure 1E). The elevated *Bifidobacterium* and its unique ABC transporters characterized in YN_ML might be attributed to the presence of raffinose and stachyose in specific pollen or nectar, which are toxic to the honeybees (Barker, 1977).

At a finer taxonomic scale, 14 of the 17 featured PTS genes had significant population-distinct SNV sites coded by SDP from *Lactobacillus* Firm-5, and 9 of the 16 ABC transporters harbored significant population-distinct SNV sites coded by SDPs from *Lactobacillus* Firm-5 and *Apibacter* (Supplementary Table 8). One featured gene *ulaC* (ascorbate PTS system EIIA or EIIAB component, K02821), coded by SDP from *Lactobacillus* Firm-5 showed significant population-distinct copy number variations (CNVs) (Supplementary Table 9). Thus, the variations in functional genes seemed to have been caused by changes in the featured bacterial composition at both phylotype and strain levels.

Besides PTS and ABC genes, six GH genes were featured in *A. cerana* populations (from GH1, GH3, GH29, GH36, GH43, and GH78 family) and were mainly contributed by *Gilliamella*, *Lactobacillus*, and *Bifidobacterium* (Figure 5B and Supplementary Table 10). To construct the profile for major gene families involved in glycoside breakdown, we used dbCAN2 (Zhang et al., 2018) to annotate all GH and polysaccharide lyase (PL) genes. We discovered that the GH/PL profiles varied across populations (Supplementary Figure 16). Additionally, the non-core bacterium also encoded novel 1265 GH genes. For instance, *Dysgonomonas* contributed unique GH gene families in *A. cerana*, including GH57, GH92, GH133, and GH144 (Supplementary Table 10). This non-core bacterium was featured in the HN_QZ population (Figure 1E), likely due to its contribution to unique GH gene sets.

Some of the six featured GH genes were positioned together with featured PTS or ABC transporters on the genome. Together, these genes formed CAZyme gene clusters (CGCs), performing sequential functions in polysaccharide degradation and transportation. For example, in *Lactobacillus* Firm-5, the featured 6-phospho-beta-glucosidase (*bglA*) from the GH1 family, PTS system genes for beta-glucoside, and cellobiose were usually clustered and formed CGCs (Figure 5C), and all these genes were enriched in the SC_AB population. In *Bifidobacterium*, the raffinose/stachyose/melibiose transport system msmEFG and alpha-galactosidase from the GH36 family involved in raffinose/melibiose degradation were usually located together (Figure 5D). These genes were all featured in the YN_ML population, which had *Bifidobacterium* as the featured phylotype.

### The feeding experiment verified the contribution of pollen polysaccharide composition to the trade-off of *Gilliamella* and *Lactobacillus* Firm-5

Our investigation of *A. cerana* guts from its natural range revealed antagonistic abundance between the two core-bacteria *Gilliamella* and *Lactobacillus* Firm-5 across geographic populations (Figure 2B). As both lineage and function diversities of honeybee gut bacteria were correlated to pollen diets (Figures 2D, 3D), we speculate that characteristic traits in local food resources may have led to bacterial community shifts observed at the grand scale. To test this hypothesis, we conducted feeding experiments to verify whether functional adaptations observed in metagenomes can lead to adaptive advantages in bacterial competition.

As the main structural components of the pollen wall, pectin and cellulose were chosen as representative nutritional contents to examine the impacts of food on the abundance variation between *Gilliamella* and *Lactobacillus* Firm-5 in co-feeding experiments. In the main gut microbe phylotypes in the honeybee, only *Gilliamella* are able to degrade the polygalacturonic acid (PGA), the backbone of pectin (Engel et al., 2012). On the other hand, cellobiose (the key metabolite of cellulose) related PTS genes (Supplementary Table 5) and the metabolic pathway (ko00500, starch, and sucrose metabolism) were highly enriched in the SC_AB population, as revealed by the metagenome data. The newly assembled *Lactobacillus* Firm-5 genome also showed elevated copy numbers in cellobiose PTS (Figure 5A). As such, we anticipated that local food with a higher proportion of pectin would increase the fitness of *Gilliamella*, and food with a higher proportion of cellulose would favor *Lactobacillus* Firm-5 in the community.

We fed *A. cerana* workers that were colonized with an equal abundance of *Gilliamella* and *Lactobacillus* Firm-5, with cellobiose, pectin, and cellulose mixture with different concentrations (1:10 and 10:1, respectively) and examined corresponding changes in bacterial composition after 4 days. Interestingly, the absolute abundance of *Lactobacillus* Firm-5 was always higher than *Gilliamella* in the control group, which was only fed with sucrose (Figures 5E–G), indicating a predominant role of *Lactobacillus* over *Gilliamella* in the given condition. The absence of pollen in food, and the absence of sucrose PTS genes in the strain we used (belonging to Gillia_Acer_2 SDP, Figure 5A) might explain the low abundance of *Gilliamella* in the control group. The absence of *Snodgrassella* might also affect the colonization of *Gilliamella* (Martinson et al., 2012; Kwong et al., 2014).

After feeding cellobiose, the absolute abundance of both *Gilliamella* and *Lactobacillus* Firm-5 significantly increased relative to the control group (Figures 5E,F), which was in accordance with the presence of cellobiose PTS genes in both phylotypes (Figure 5A). As expected, *Gilliamella* and *Lactobacillus* Firm-5 showed different responses to the mixed food with varied concentrations of pectin and cellulose. The absolute abundance of *Gilliamella* did not show significant variation after feeding food of pectin:cellulose (1:10), but the abundance of *Lactobacillus* Firm-5 significantly increased (Figures 5E,F). On the other hand, the absolute abundance of *Gilliamella* showed a significant increase after feeding food of pectin:cellulose (10:1), but the abundance of *Lactobacillus* Firm-5 did not vary significantly (Figures 5E,F). The varied proportion of pectin and cellulose impacted the antagonistic of *Gilliamella* and *Lactobacillus* Firm-5. These results suggested that diet, pollen polysaccharide, in particular, is an important factor in shaping gut bacterial composition and functions in *A. cerana*.

## Discussion

### The progressive change of gut microbiome in Asian honeybee populations

In this study, we carried out comprehensive investigations on the gut microbiomes of the widespread Asian honeybee *A. cerana* at the population level. While many studies have contributed to our knowledge of the honeybee gut microbiota, little is understood about how this essential symbiont system evolves with the host. In agreement with previous studies on both *A. mellifera* (Rothman et al., 2018; Ellegaard and Engel, 2019) and *A. cerana* (Ge et al., 2021), our results revealed variations in gut microbes among *A. cerana* individuals, even among those from the same hive. The intra-colony variation might be related to differed social interactions among honeybee individuals (Powell et al., 2014) or varied exposure to the stored pollens and other hive materials (Anderson et al., 2022) in the honeybee.

Our studied system involved 15 geographic populations of *A. cerana*, and we were particularly interested in understanding gut variations among the seven genetically distinctive populations, which we showed in our recent study (Ji et al., 2020) that had diverged genetically and morphologically at a subspecies level. These subspecies have been confined to drastically different habitats (e.g., mountain valleys of >3,000 m, tropical islands, temperate plains, hills, etc.). In contrast to the abrupt distinction between *A. mellifera* and *A. cerana*, the gut microbiome of honeybee populations showed progressive change within *A. cerana* (Supplementary Figure 17). The bacterial compositions across populations showed significant variations at phylotype, SDP, strain, and gene content levels, albeit with extensive overlaps. The strain composition of *Gilliamella* and *Snodgrassella* was largely similar among populations of western honeybees from four different states in the United States (Bobay et al., 2020). The gut microbiota community from 18 different human populations across geography also showed extensive overlap (Smits et al., 2017), implying a common trend in gut microbiome evolution for hosts exhibiting a continuous and wide-range distribution.

In the western honeybee, host genetics influenced the gut microbe composition, where *Bifidobacterium* abundance was associated with the genotype of the host glutamate receptor (Wu et al., 2021). Different from Wu et al. (2021), the complex background heterogeneity combined with a limited sample size might have masked apparent host genetic influence on gut bacteria at the local scale in our study, which may explain the weak signal reported in our GWAS analysis. However, our findings genuinely reflect the host genetic background and associated microbiota, which could not have been discovered without a broad scale sampling. With an in-depth understanding of the molecular mechanisms underlying honeybee host-gut bacteria interactions in the future, we expect that a more focused genomic screening on these target genes would reveal their specific contributions in widely distributed native bee populations.

### Gut microbiome evolution under local diet shift in Asian honeybee

The honeybees consume relatively simple but consistent food, i.e., pollen and honey, and pollen is especially important to the gut microbes. Controlled experiments on the diet with or without pollen influenced the total and specific gut bacteria abundance (Kešnerová et al., 2020; Ricigliano and Anderson, 2020). Pollen diet also facilitates the co-existence of closely related *Lactobacillus* species by using different pollen-derived carbohydrate substrates (Brochet et al., 2021). Although controlled experiments conducted on *A. mellifera* have built the foundation on diet influence on honeybee gut microbiota, we knew little about how natural diets influenced honeybee gut microbiota in their native range.

Floral shifts are a common theme during range expansion and habitat adaptation of the honeybees (Ji et al., 2020). The change of locally flowering plants inevitably alters nutrients for honeybees and the associated gut microbiome, because nutritional components vary in both pollen and nectar across plant species. Pollen walls are enriched in polysaccharides in the forms of cellulose, hemicelluloses, and pectin, which serve as major food resources for the gut bacteria (Engel et al., 2012; Zheng et al., 2019). Previous studies have shown the contents of cellulose and hemicellulose varied in pollen of different species (McLellan, 1977) and in bee pollen collected from different regions (Herbert and Shimanuki, 1978). Similarly, sugar composition in nectar varied among flowers (Chalcoff et al., 2006). Particularly, nectar may contain low doses of sugars that are toxic to the honeybee, such as raffinose and mannose (Barker, 1977). Thus, both the general floral configuration and specific flower traits could serve as determining factors for the formation of a local honeybee gut profile.

Our recent work on the evolution of mainland *A. cerana* revealed that the changing floras led to a convergent adaptation of the honeybee (Ji et al., 2020). Here, we showed that both microbial composition and function of the honeybee gut microbiota exhibited progressive change throughout the studied natural range. The variation could be partially explained by the pollen diet, which is closely related to changing flora in the habitat. Such an intra-species transition in the gut microbiome reflects the evolutionary consequence of collective adaptation of both the honeybee and its symbionts.

Besides amino acids, lipids, and vitamins, pollen is a source of diverse carbohydrate sources. Carbohydrate metabolism is the second most abundant functional class of bacterial transcripts (Lee et al., 2015). Different honeybee gut bacteria species showed varied GH transcripts (Lee et al., 2015) and activities (Ricigliano et al., 2017). The PTS and ABC transporters, genes involved in the transportation of multiple types of polysaccharides, were also associated with different gut bacteria species (Lee et al., 2015). Accordingly, the PTS and ABC transporters were primarily encoded by *Gilliamella* and *Lactobacillus* Firm-5, representing the most enriched transporters among all bacterial genes featured in local populations of *A. cerana*. Our feeding and inoculation assays further showed that pollen polysaccharides determined the abundance of the two core bacteria, *Gilliamella* and *Lactobacillus* Firm-5. The role of core bacteria in local adaptations was reinforced by evidence showing their dominant contributions to genes related to pollen and nectar digestions.

Unexpectedly, non-core bacteria sometimes became abundant in local honeybee populations. For instance, *Dysgonomonas* was typically low in abundance among *A. cerana* individuals, as reported in both *Apis nigrocincta* (Lombogia et al., 2020) and *A. mellifera* (Erban et al., 2017). But this bacterium contributed a series of unique GH genes in FJ_FZ and HN_QZ populations, thereby becoming abundant and common in the corresponding gut microbiome. This observation suggested that local food resources might trigger bacterial species turnover when non-core bacteria became more suited to new diets, which again highlights the significance of diet on the gut profile.

### Population heterogeneity needs to be considered for the evolution and adaptation of honeybee microbiomes

A recent study suggested that both lineage and function diversities of the gut microbes were significantly lower in *A. cerana* when compared with *A. mellifera* (Ellegaard et al., 2020). However, this conclusion was drawn based on two *A. mellifera* colonies from Switzerland, two colonies of both *A. mellifera* and *A. cerana* from two sites in Japan, it is difficult to anticipate whether such a distinct pattern could be generalized when population gradients of both honeybee species are taken into consideration. Although the present study was not designed for comprehensive analyses of inter-species comparisons, our results provided insights into how intra-species variations in gut microbiota might affect interpretations of differences between honeybee species.

Although the per-bee gene diversity was generally lower in *A. cerana* microbiota than *A. mellifera*, individual bees of different *A. cerana* populations showed variation (Supplementary Figure 18A). In addition, the divergence of the accumulated gene diversity between the two species was much less significant than previously suggested. The Japanese populations representing *A. cerana* in the earlier study (Ellegaard et al., 2020) were one of the least variable populations among all *A. cerana* populations investigated in this study (Supplementary Figure 18B). Given the large variations observed among *A. cerana* populations, it is unknown whether a similar difference might also be common within *A. mellifera* and how that might influence the distinctions between these two widely distributed honeybee species. Additionally, other confounding factors should also be taken into consideration to gain a comprehensive understanding of honeybee gut microbiomes. In particular, the evolutionary pathways and phylogenetic relationships of focal populations, the specifics in honeybee management (such as colony merging and artificial diet additions), and other human interventions, may all have significant impacts on the honeybee gut profile. As the gut symbiont profile is a signature of the natural adaptation of the host to specific habitats, it would seem that comparisons between microbiomes of intra- and inter-host honeybee species should always be placed in a context of specific environments.

Since worker age (Hroncova et al., 2015) and seasonality (Almeida et al., 2022) showed effects on honeybee gut microbiome, these factors need to be considered in the data interpretation. Additionally, the limited sampling for each local population might also under-estimate the gut microbe diversity and bring bias into intra-colony variation (Rothman et al., 2018). However, season control and simultaneous age marking are admittedly difficult for sampling honeybees from a wide geographic range. In our study, we chose a practical method to specifically sample nurse bees based on their morphology. Admittedly, such criteria are not as accurate as individual marking and errors are possible. Despite the potential influence of seasonality, populations sampled in the same month did not show any elevated affinity to those sampled from different months (Figure 2 and Supplementary Figure 14). Nevertheless, with honeybee age and season controlled, collecting enough individual bees at the intra- and inter- colony levels could improve future research initiatives on investigations of native honeybee colonies. Notably, season control is to collect bees with the same circadian activity and probably not the same month for different populations located across temperate and tropical regions.

Our study took a first step toward understanding the relative contribution of diet and host genetics on the gut microbiota of widely ranged honeybee populations. Our results detected localized gut features at both species and functional levels throughout the distribution range. However, the gut microbiome showed unexpected extensive overlap across the investigated ranges, which covered temperate to tropical regions. These results suggested that progressive change is the foundation of gut microbiome evolution in the Asian honeybee and specialized bacterial traits help to adapt to local diets. In this regard, regional floral diversity could serve as a key to maintaining characteristic repertoires of honeybee gut microbes, which is tremendously important for honeybee health as a whole. Therefore, a sustaining plant community containing diverse endemic flower species should be considered a key part of a honeybee conservation plan. On the other hand, the fitness of gut microbiomes of the honeybee populations may play an unforeseen role in the survival of colonies, during honeybee introduction, hybridization, and especially translocation.

## Data availability statement

The datasets presented in this study can be found in online repositories. The names of the repository/repositories and accession number(s) can be found below: https://www.ncbi.nlm.nih.gov/, PRJNA705951.

## Author contributions

XiZ designed the study. XiZ, SL, and XeZ organized and coordinated the study. QS coordinated sample collection, bacterial isolation, and genome annotation, reference-based metagenome mapping, and SDP analysis. MT conducted *de novo* assembly of bacteria and metagenome and metagenomics analysis. SL conducted genetic variation analysis of *A. cerana*, heritability, and GWAS analysis. JH conducted diet profiling. QS and JT conducted feeding experiments and qPCR assay. XiZ, QN, and XL organized sampling. SL, XiZ, and XgZ wrote the first draft. All authors contributed to the article and approved the submitted version.

## Footnotes

1
https://github.com/BGI-shenzhen/BamDeal

2
http://broadinstitute.github.io/picard/

3
https://github.com/widdowquinn/pyani

4
https://ftp.ncbi.nlm.nih.gov/genomes/refseq/

5
https://github.com/aquaskyline/SOAPcoverage

6
https://www.kegg.jp/kegg/tool/map_pathway2.html
