# Supplementary material for: Significant compositional and functional variation reveals the patterns of gut microbiota evolution among the widespread Asian honeybee populations
Source: Front Microbiol. 2022 Sep 2;13:934459. doi: 10.3389/fmicb.2022.934459 (PMC9478171; doi:10.3389/fmicb.2022.934459)
Supplement: Supplementary file 1 [file Data_Sheet_1.zip › supplements_20220812/SOM_20220812-Frontiers.docx]

**Supplementary Figures**


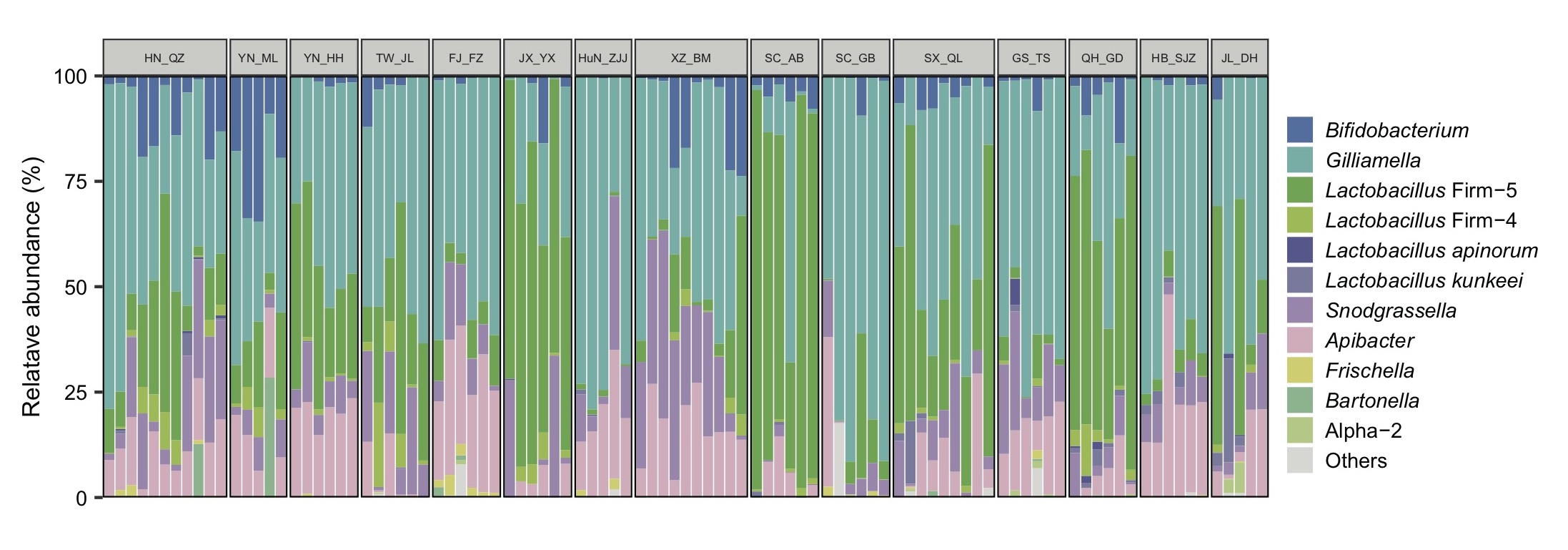


**Supplementary Figure 1. Community composition and diversity at the phylotype level in gut microbiota from *A. cerana*.** The results were analyzed by Kraken2 with 390 bee gut bacteria genomes as reference in Supplementary Table 2. Host populations are indicated by short bars at the top.


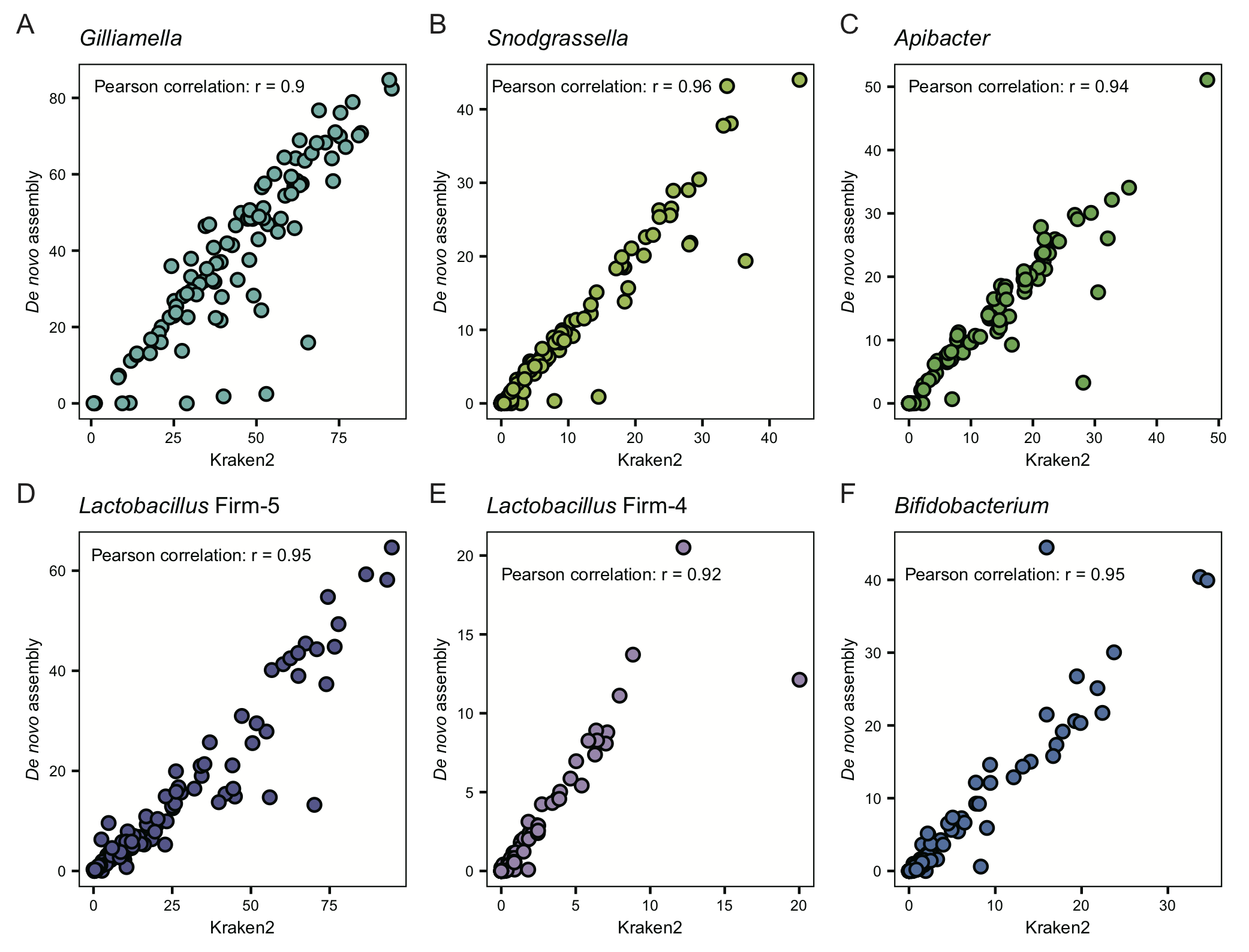


**Supplementary Figure 2. The relative abundance of core bacteria with the *de novo* methods were highly correlated with the results from reference-based Kraken2 methods.**


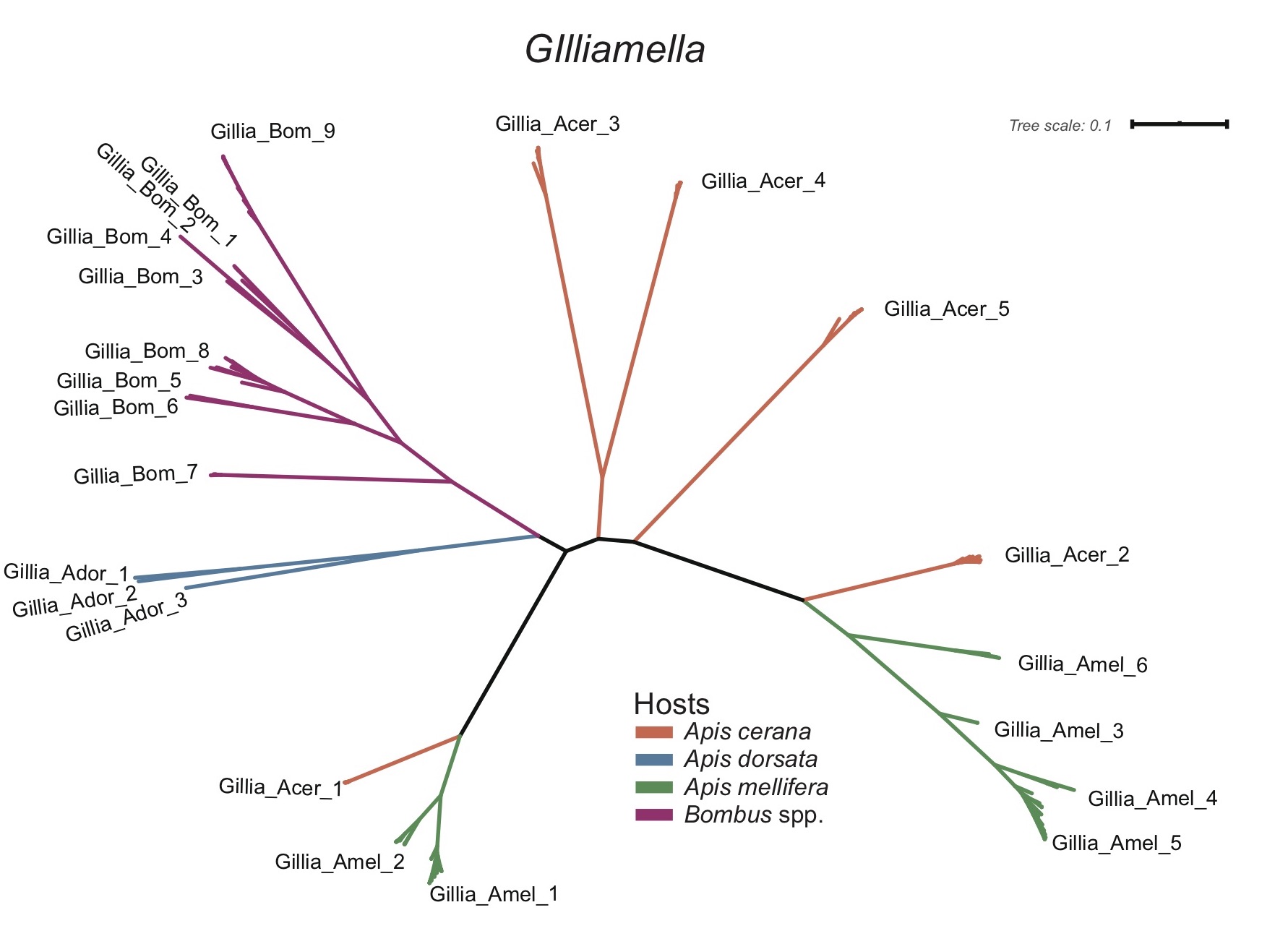


**Supplementary Figure 3. Maximal-likelihood (ML) phylogenetic tree of *Gilliamella* strains from bees.** ML tree was built on the core genes existing in all strain genomes. Branch colors correspond to host species.


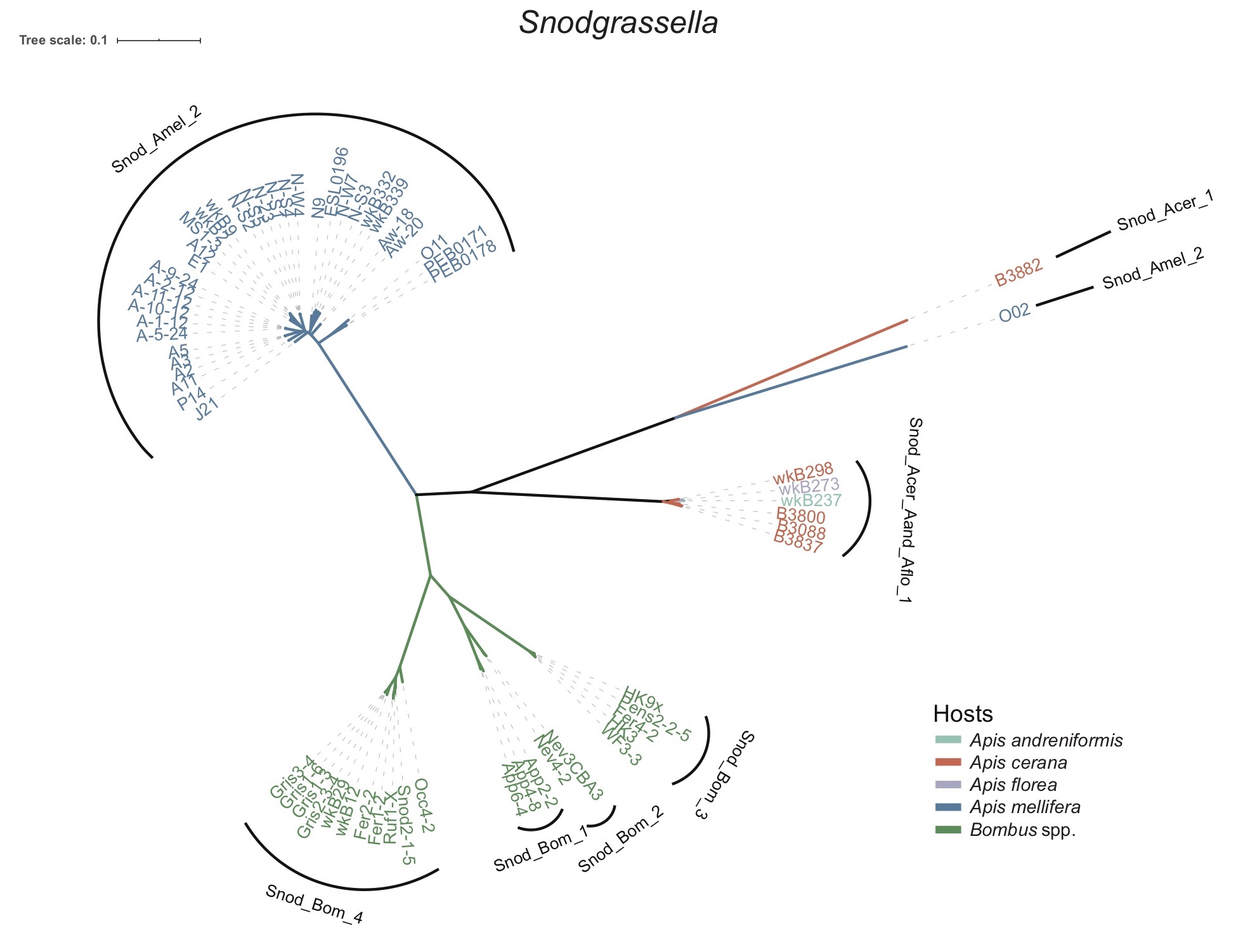


**Supplementary Figure 4. Maximal-likelihood (ML) phylogenetic tree of *Snodgrassella* strains from bees.** ML tree was built on the core genes existing in all strain genomes. Branch colors correspond to host species.


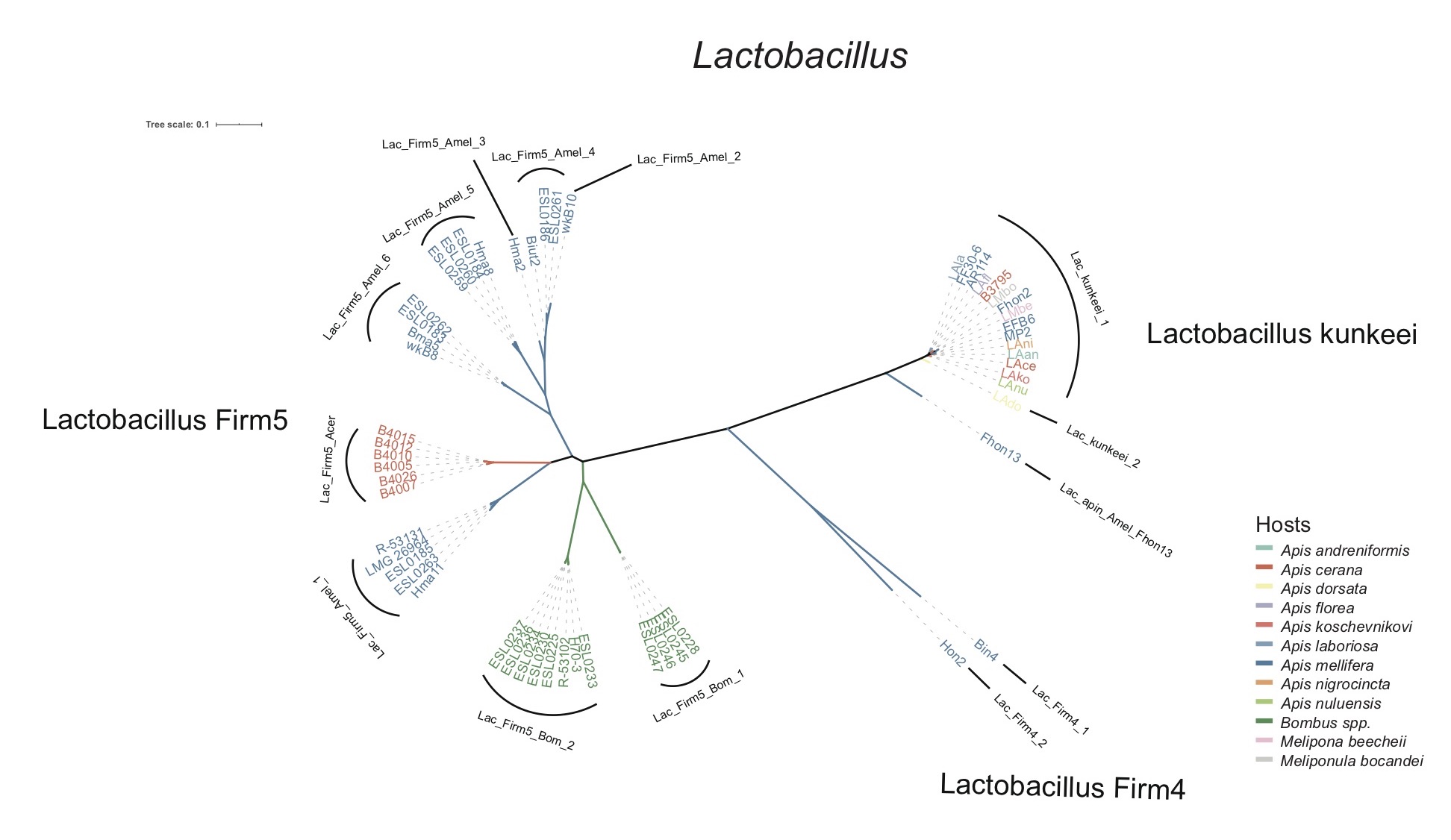


**Supplementary Figure 5. Maximal-likelihood (ML) phylogenetic tree of *Lactobacillus* strains from bees.** ML tree was built on the core genes existing in all strain genomes. Branch colors correspond to host species.


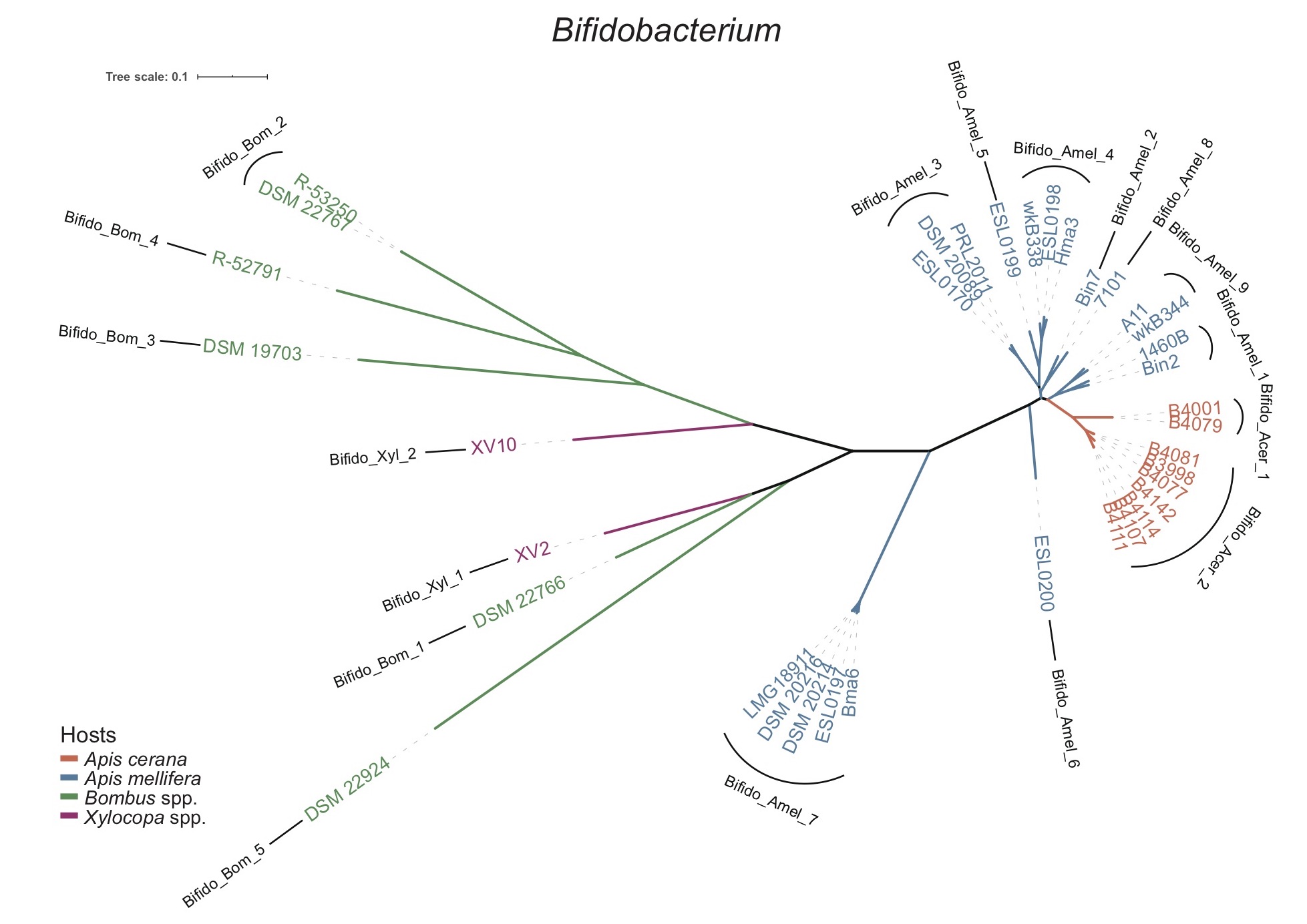


**Supplementary Figure 6. Maximal-likelihood (ML) phylogenetic tree of *Bifidobacterium* strains from bees.** ML tree was built on the core genes existing in all strain genomes. Branch colors correspond to host species.


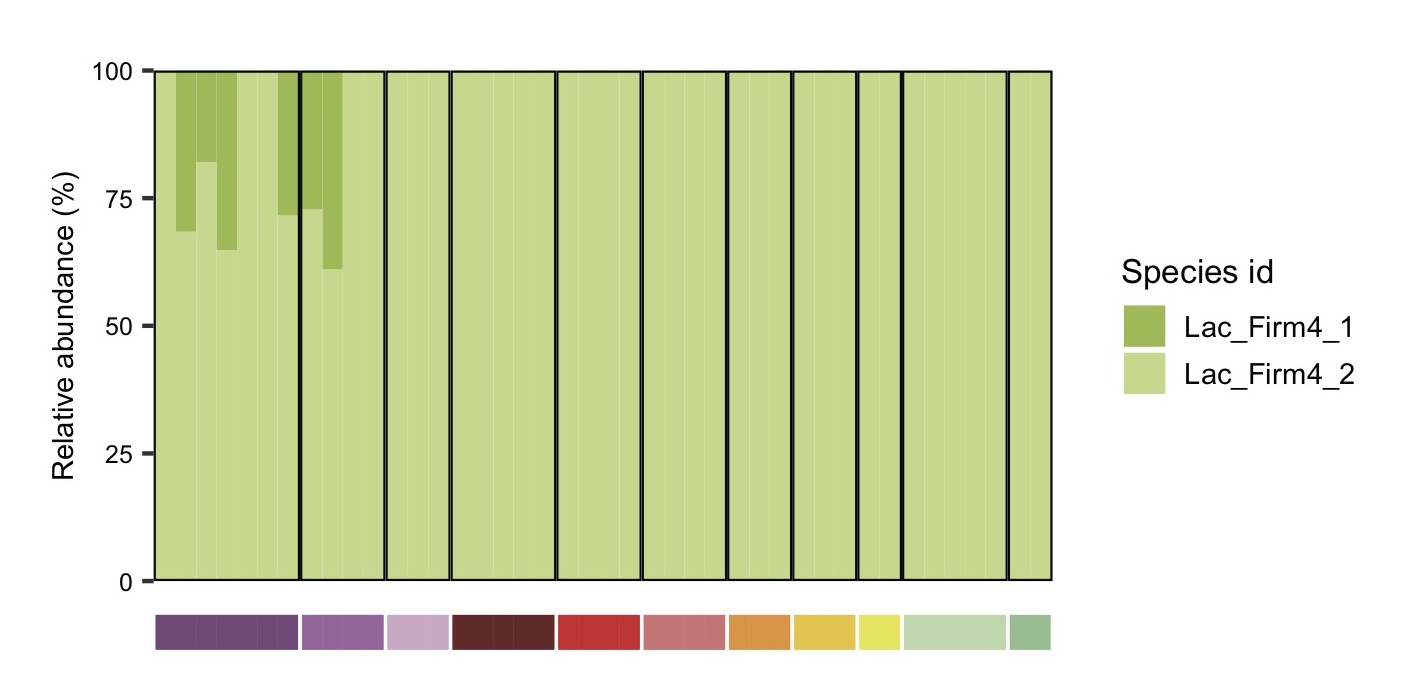


**Supplementary Figure 7. The SDP composition of *Lactobacillus* Firm-4 in gut metagenome samples of *A. cerana* estimated by MIDAS.**


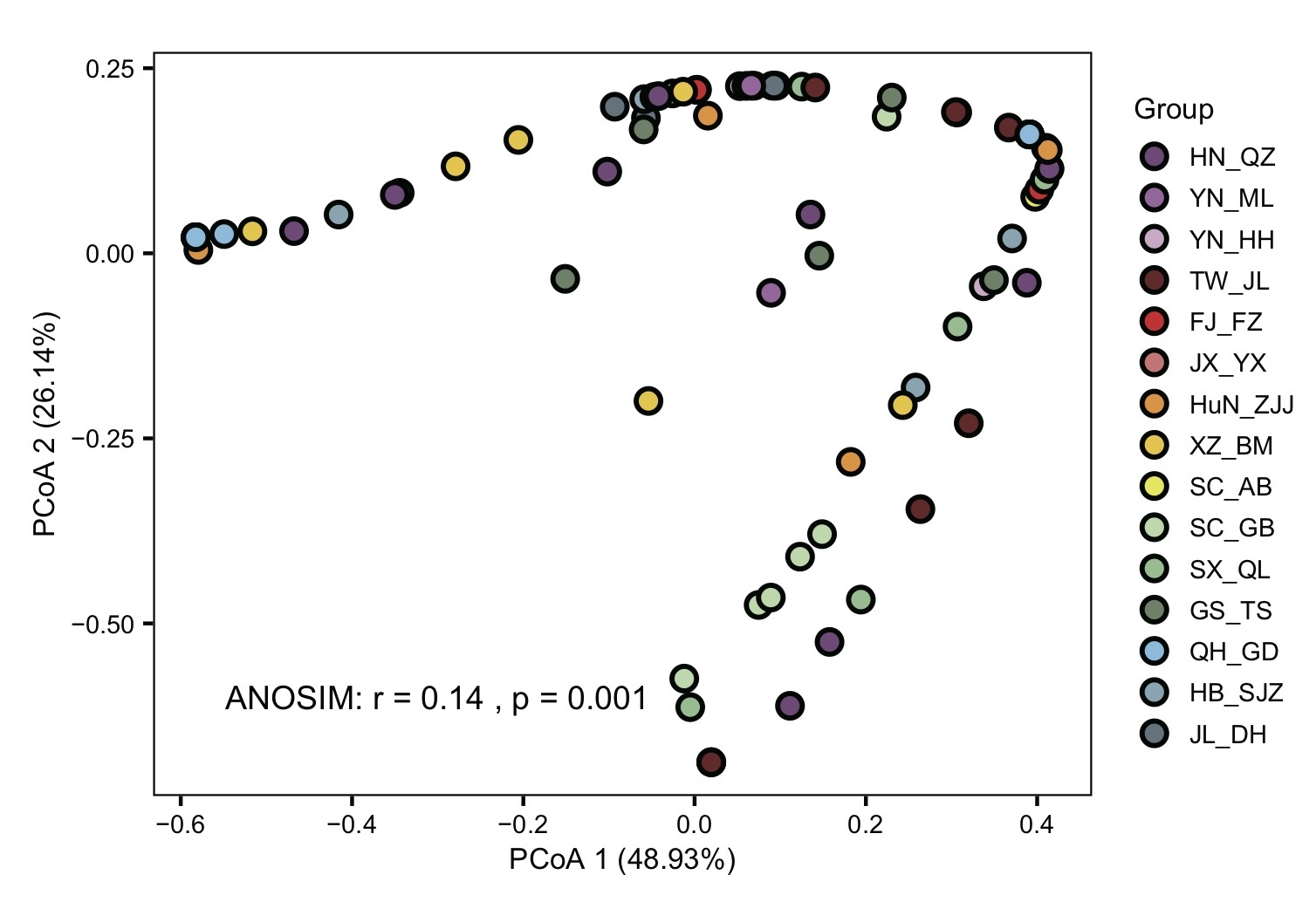


**Supplementary Figure 8. PCoA of *Gilliamella* SDP composition in populations of *A. cerana*.**


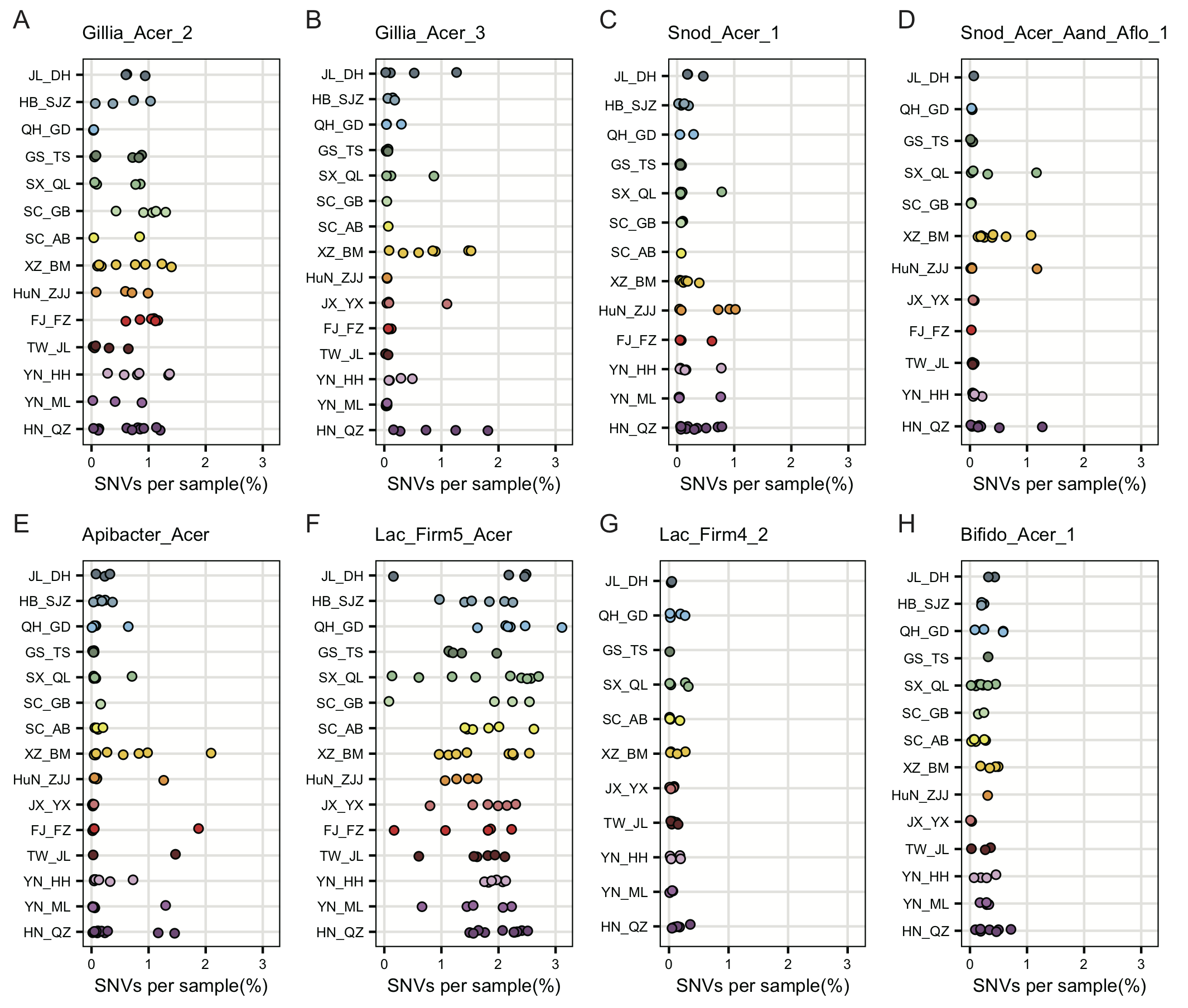


**Supplementary Figure 9. Fraction of SNVs for the dominant SDPs varied in populations of *A. cerana*.** Dominant SDPs include those show relatively high frequency in all samples. SNVs in each sample were calculated from all SNPs existing in each SDP.


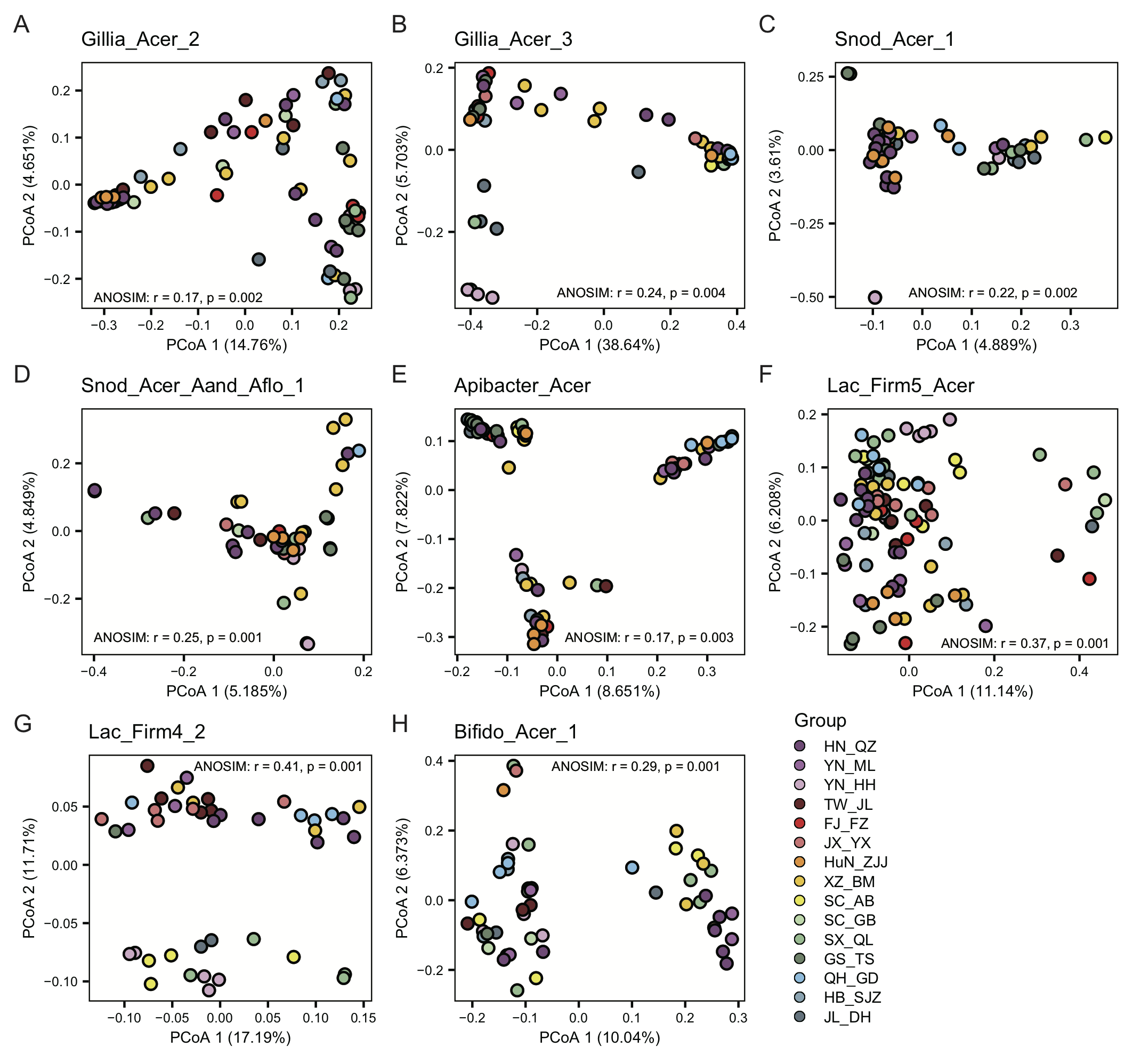


**Supplementary Figure 10. SNV differences for dominant SDPs in the gut microbiome from 15 populations of *A. cerana*.**


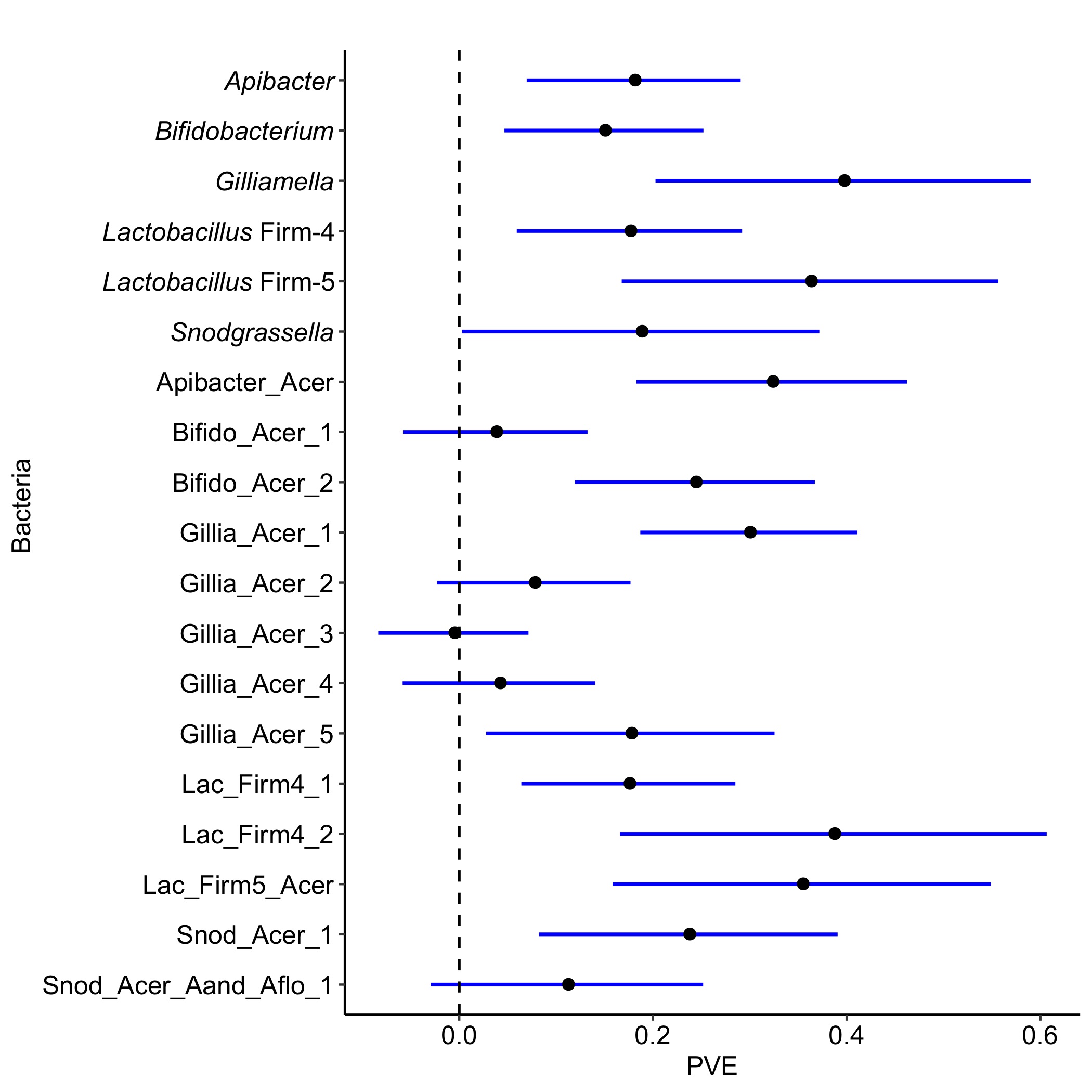


**Supplementary Figure 11. SNP heritability of the core phylotypes and SDPs for gut microbiota in *A. cerana*.** Each point represents the estimated PVE with GEMMA by genome-wide SNPs for the abundance of the core phylotypes and SDPs. Bars indicate SE measurements around the estimate. PVE: Percentage of Variance Explained.


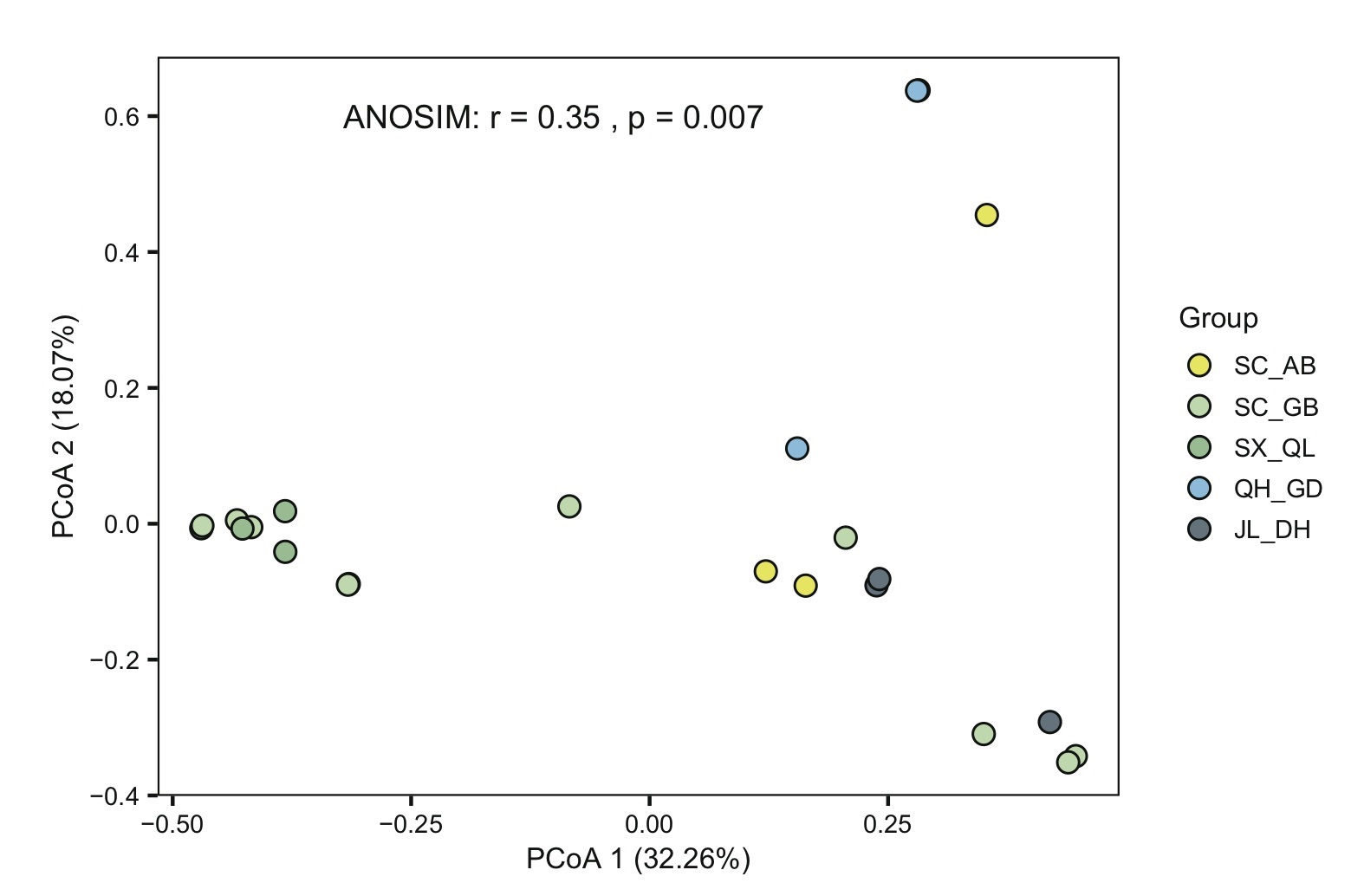


**Supplementary Figure 12. The pollen composition at the family level varied in honey from populations of *A. cerana***


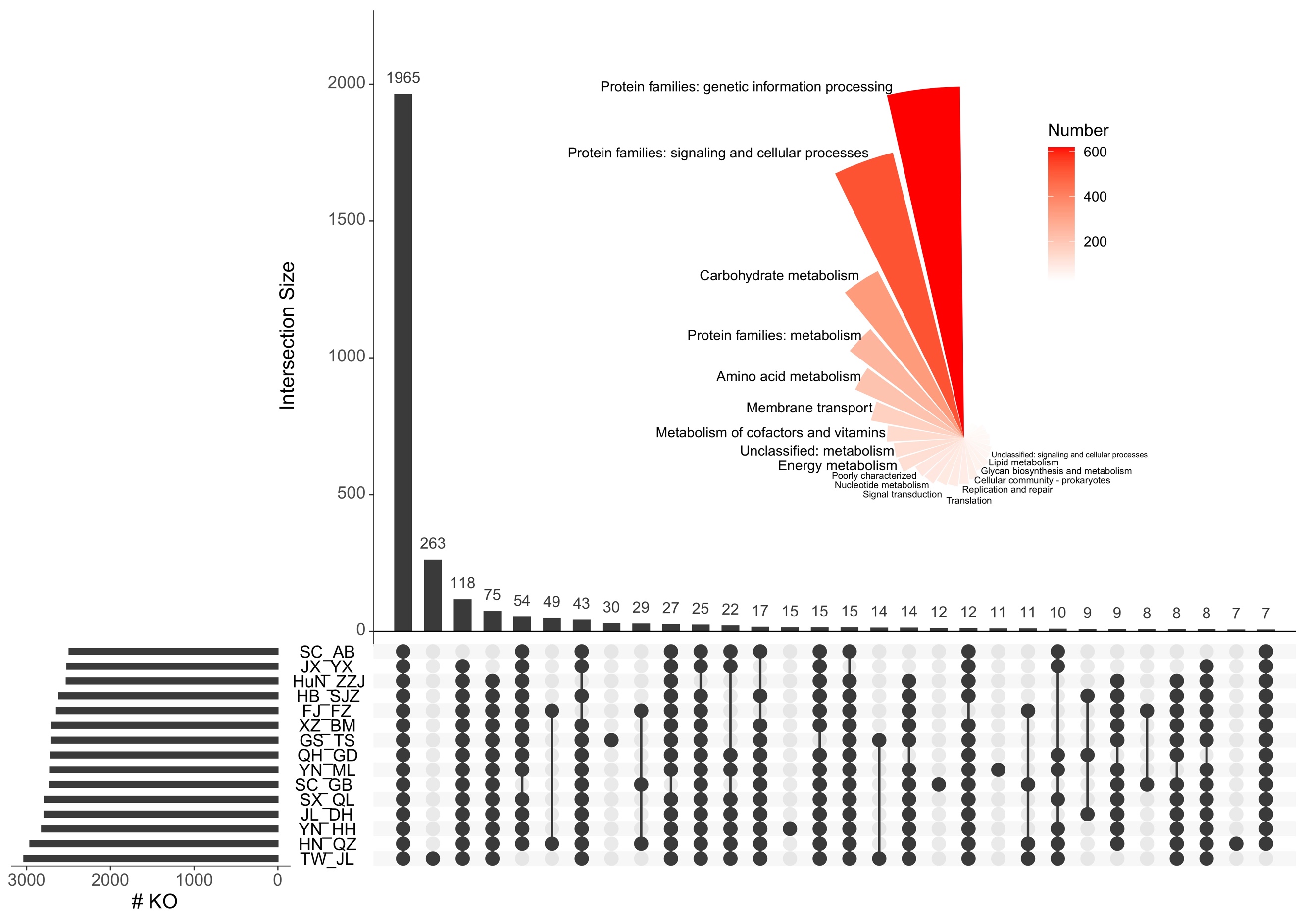


**Supplementary Figure 13. Distribution of KEGG orthologs (KOs) in gut microbes from 15 populations of *A. cerana*.** Horizontal bars on the bottom left represent KO numbers annotated in each population. Vertical bars indicate the numbers of KOs exclusively associated with one or multiple groups. The common KO numbers in each category are in red. The area of each KO category is proportional to the KO number.


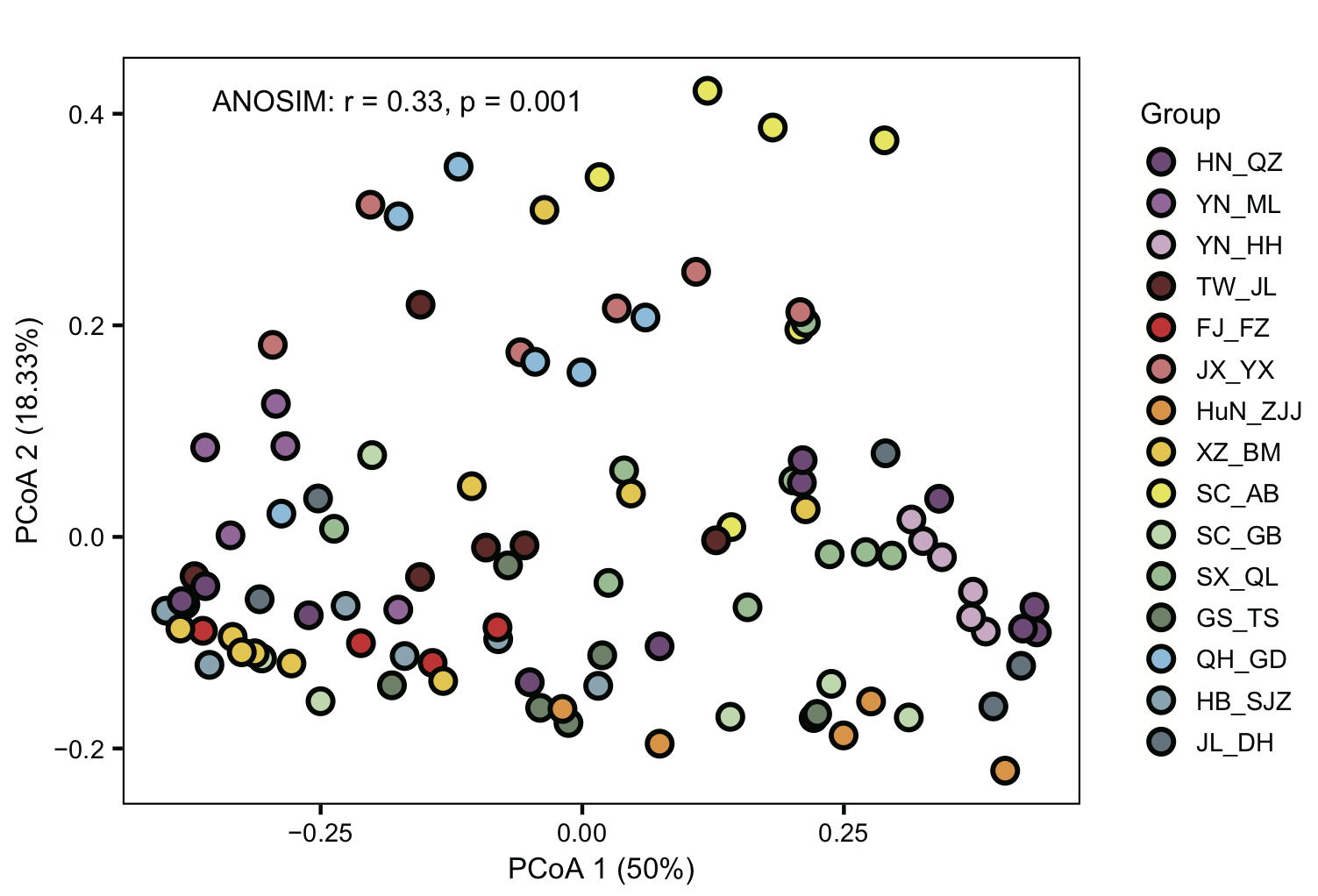


**Supplementary Figure 14. Gut microbe KO abundance showed significant difference among populations with PCoA.**


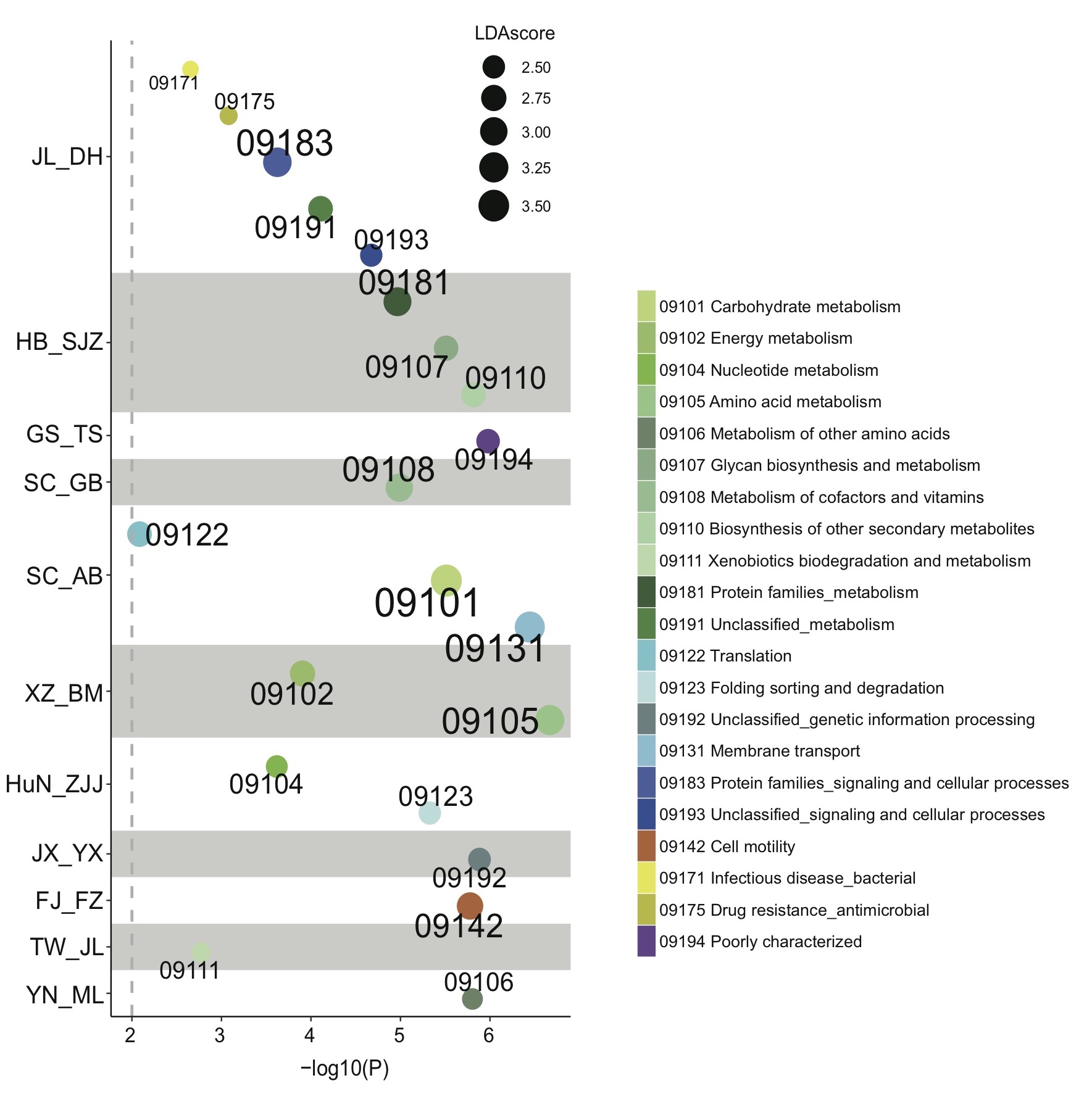


**Supplementary Figure 15. Characterized KEGG categories in gut microbiota from populations of *A. cerena* using LEfSe algorithm.** The size of the squares represents the LDA score.


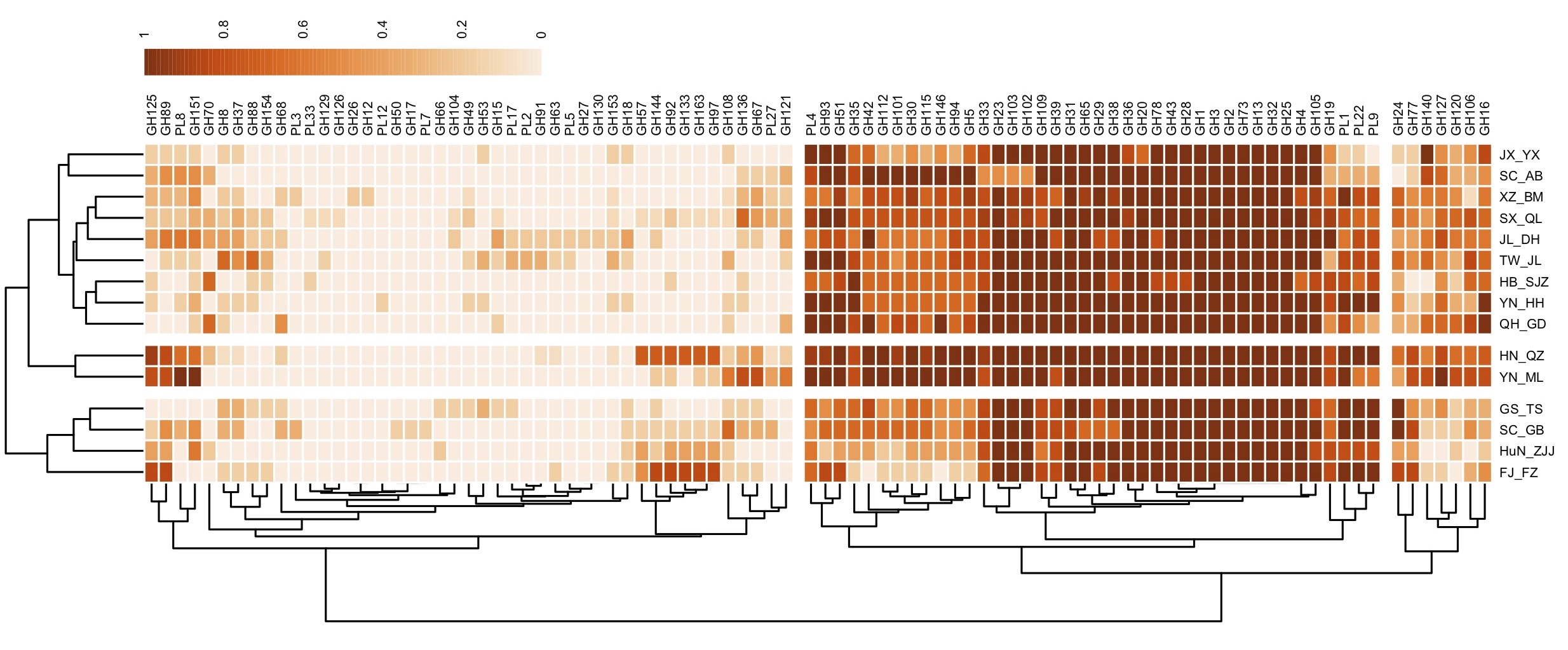


**Supplementary Figure 16.** **Glycoside hydrolases (GH) and polysaccharide lyases (PL) gene profiles in gut microbiota from populations of *A. cerana.*** Color represents the frequency of the GH/PL family in each population.


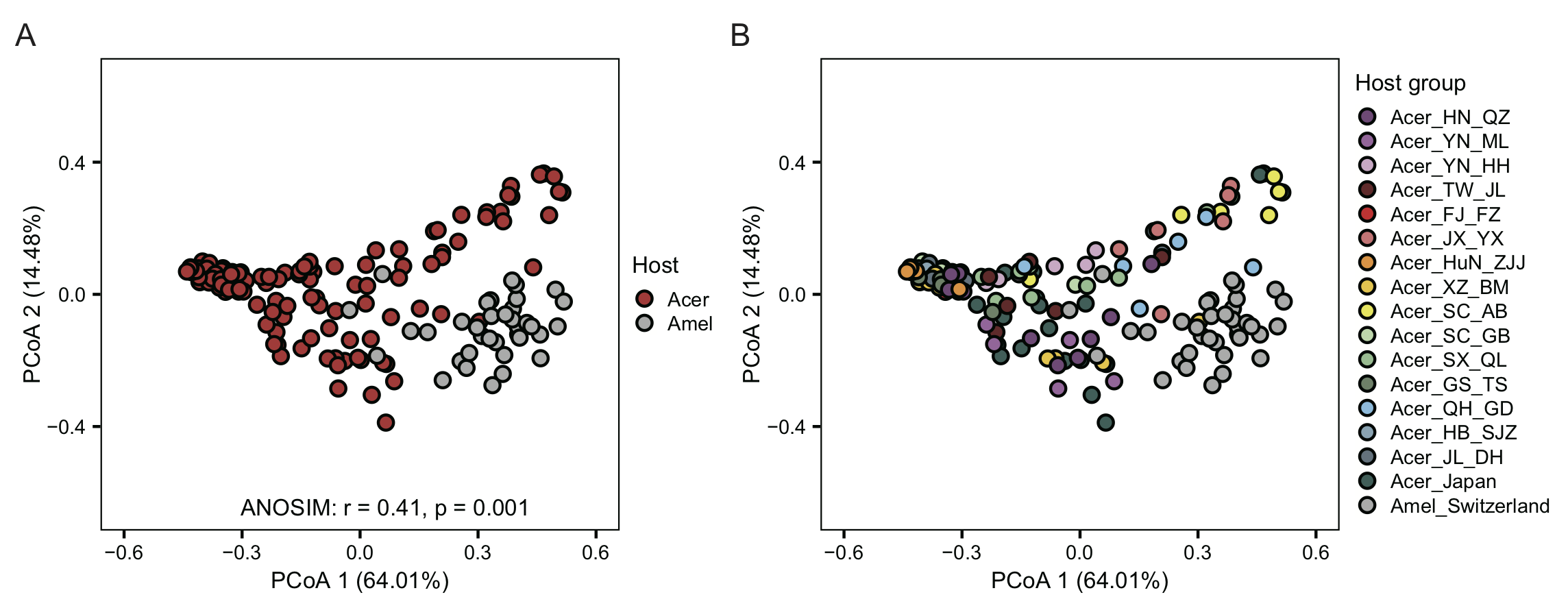


**Supplementary Figure 17. Gut microbe community composition showed significant difference between *A. cerana* and *A. mellifera* with PCoA.** (A) Gut microbe community difference at the host species level. (B) Gut microbe community composition showed variation among different populations of *A. cerana* (including population from Japan) with PCoA.


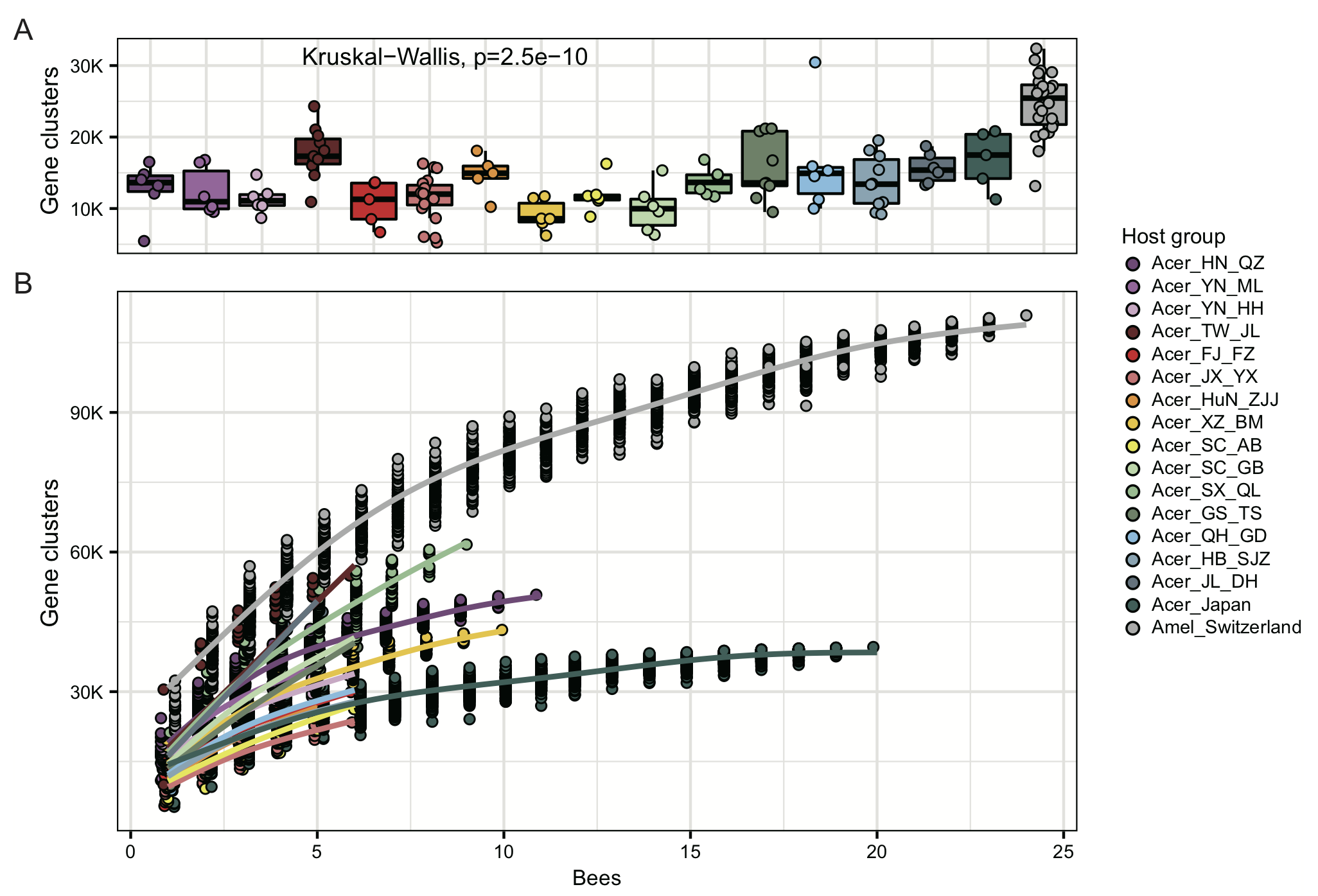


**Supplementary Figure 18. Gene cluster numbers in gut microbiota varies in *A. cerana* and *A. mellifera*.** All comparisons were based on the 400Mb bacteria-mapped reads. (A) Gene cluster numbers per sample showed difference in different populations of *A. cerana* and *A. mellifera*. (B) Cumulative number for gene cluster numbers in gut metagenome from *A. mellifera* and each population of *A. cerana*.
